# Magnetic Resonance Spectroscopy of Cystathionine and 2‐Hydroxyglutarate in Brain Tumors

**DOI:** 10.1002/nbm.70181

**Published:** 2025-11-25

**Authors:** Changho Choi, Mai Huynh, Zoltan Kovacs, William J. Behof, Wellington Pham, Sandeep K. Ganji, Zhongxu An, Toral R. Patel, Elizabeth A. Maher, Julia D. Berry, Bret C. Mobley, Larry T. Davis, Colin D. McKnight, Sumit Pruthi, Ryan T. Merrell, Alexander C. Mohler, Patrick D. Kelly, Reid C. Thompson

**Affiliations:** ^1^ Vanderbilt University Institute of Imaging Science Vanderbilt University Medical Center Nashville Tennessee USA; ^2^ Department of Radiology and Radiological Sciences Vanderbilt University Medical Center Nashville Tennessee USA; ^3^ Advanced Imaging Research Center University of Texas Southwestern Medical Center Dallas Texas USA; ^4^ Philips Cambridge Massachusetts USA; ^5^ Department of Neurological Surgery University of Texas Southwestern Medical Center Dallas Texas USA; ^6^ Department of Internal Medicine University of Texas Southwestern Medical Center Dallas Texas USA; ^7^ Department of Pathology, Microbiology and Immunology Vanderbilt University Medical Center Nashville Tennessee USA; ^8^ Department of Neurology Vanderbilt University Medical Center Nashville Tennessee USA; ^9^ Department of Neurological Surgery Vanderbilt University Medical Center Nashville Tennessee USA

**Keywords:** 1p/19q codeletion, 2‐hydroxyglutarate, 3 T, cystathionine, IDH (isocitrate dehydrogenase) mutation, magnetic resonance spectroscopy, PRESS (point‐resolved spectroscopy)

## Abstract

Codeletion of chromosome 1p and 19q arms in a subset of gliomas has been shown to associate with the accumulation of cystathionine in the tumor. Here, we report the analyses of cystathionine and 2‐hydroxyglutarate (2HG) in 38 biopsy‐proven glioma patients, as measured with TE 97‐ms point‐resolved spectroscopy (PRESS) at 3 T. Following the confirmation of in‐house calculated cystathionine basis signals in a phantom solution, LCModel spectral fitting was performed to decompose metabolite signals, and the millimolar concentrations of metabolites were estimated with reference to water. Cystathionine was estimated to be significantly higher in IDH mutated 1p/19q codeleted gliomas than in noncodeleted gliomas (1.4 ± 1.1 vs. 0.3 ± 0.4 mM; *n* = 15 and 14; *p* = 0.002). 2HG estimation was significantly higher in IDH‐mutant gliomas compared to IDH‐wildtype gliomas (3.7 ± 3.3 vs. 0.1 ± 0.1 mM; *n* = 29 and 9; *p* = 0.002). Using cutoff values from receiver operating characteristic curve analysis, the sensitivity and specificity of the cystathionine measures with respect to 1p/19q status were estimated to be 0.8 and 0.79 (cutoff at 0.6 mM), and those of 2HG measures with respect to IDH status were both unity (cutoff at 0.5 mM). A TE 113‐ms PRESS sequence was designed to improve detection of the cystathionine C4‐proton resonance at 2.72 ppm. Data from two glioma patients showed that cystathionine can be detected with minimal interference from the overlapping aspartate signal. 2D chemical‐shift imaging of cystathionine using the TE 113‐ms PRESS is demonstrated. Our MRS data confirm elevation of cystathionine in 1p/19q codeleted gliomas, as demonstrated in prior studies, and suggest that relatively high estimates of cystathionine may provide a biomarker of 1p/19q codeleted gliomas.

## Graphical abstract

Cystathionine and 2‐hydroxyglutarate were evaluated in 38 glioma patients with TE 97ms PRESS at 3T. Cystathionine level was significantly higher in IDH‐mutant 1p/19q‐codeleted gliomas than in IDH‐mutant non‐codeleted gliomas (*p* = 0.002). The sensitivity and specificity of the cystathionine measures with respect to 1p/19q status were 0.8 and 0.79, while those of 2‐hydroxyglutarate measures with respect to IDH status were both unity. Our data suggested that relatively high estimates of cystathionine may be predictive of 1p/19q codeleted gliomas.

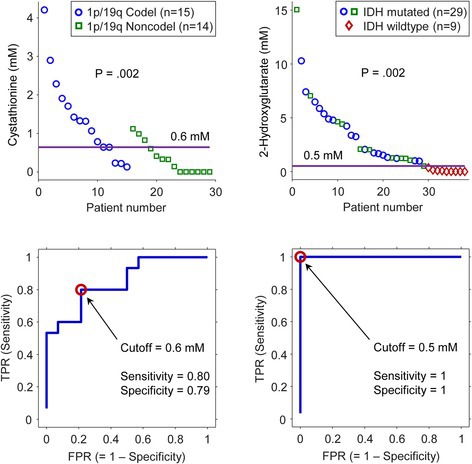

**Abbreviations**
2HG: 2‐hydroxyglutarate
AUC: area under the curve
CST: cystathionine
FOV: field of view
FPR: false positive rate
FWHM: full width at half magnitude
IDH: isocitrate dehydrogenase
NAA: N‐acetylaspartate
NAAG: N‐acetylaspartylglutamate
PRESS: point‐resolved spectroscopy
RF: radio frequency
ROC: receiver operating characteristic
SNR: signal‐to‐noise ratio
T2‐FLAIR: T2‐weighted fluid attenuated inversion recovery MRI
tCho: total choline (= glycerophosphocholine + phosphocholine)
tCr: total creatine (= creatine + phosphocreatine)
tNAA: total NAA (= N‐acetylaspartate + N‐acetylaspartylglutamate)
TPR: true positive rate
VOI: volume of interest

## 1 Introduction

Genetic alterations in isocitrate dehydrogenase (IDH) enzymes and chromosome 1p and 19q arms are centrally important in glioma characterization and treatment planning [1, 2]. Molecular tests for determining the IDH and 1p/19q mutational statuses are therefore commonly performed in biopsy analysis in clinical practice. IDH mutation occurs with metabolic reprogramming that causes accumulation of 2‐hydroxyglutarate (2HG) [3]. As a result, 2HG, which is normally present in minute quantities in the human brain, is elevated to millimolar levels. With this 2–3 orders‐of‐magnitude difference in 2HG level between IDH‐mutant and IDH‐wildtype tumors, MRS detection of 2HG can identify IDH mutation noninvasively [4, 5, 6].

In recent years, elevation of cystathionine in IDH‐mutant 1p/19q codeleted gliomas has been reported, as measured with mass spectrometry in glioma tissue as well as in vivo MRS in glioma patients [7, 8]. Cystathionine is synthesized from homocysteine in the transsulfuration pathway and may accumulate as a result of the downregulation of phosphoglycerate dehydrogenase and cystathionine gamma‐lyase in 1p/19q codeleted gliomas.

Cystathionine has eight nonexchangeable J‐coupled protons, giving multiplets approximately at five locations (3.94, 3.85, 3.11, 2.72, and 2.16 ppm) [9]. The resonances of cystathionine are present in the proximity of abundant resonances of other metabolites and macromolecules. The C4‐proton resonance at 2.72 ppm, which may be the strongest among the five resonances, overlaps with the C3‐proton resonance of aspartate [10]. Thus, detection of the presumably small cystathionine signals by conventional, short‐TE MRS may not be straightforward. The 3.11 ppm (^6^CH_2_) and 2.72 ppm (^4^CH_2_) proton resonances, which are coupled, respectively, to 3.94‐ and 2.16‐ppm resonances, appear to be good candidates in spectrally edited MRS, the latter of which was utilized in prior studies [7, 9]. TE 97‐ms PRESS, which is widely used for assessing 2HG in brain tumor patients [11, 12], was used for evaluating cystathionine in glioma patients in prior studies [8, 13]. Recently, a TE 45‐ms PRESS scheme was proposed for the detection of cystathionine in glioma patients [13]. The study reported measurements of cystathionine with reduced interference from overlapping aspartate resonances and higher sensitivity and specificity of the cystathionine measures with respect to 1p/19q status, compared with TE 97‐ms PRESS.

The capability of a single MRS protocol to precisely detect multiple clinically relevant brain metabolites could prove useful in the clinical management of brain tumors. Since the 2HG‐optimized PRESS TE 97‐ms method was published many years before cystathionine was proposed as a potential biomarker of 1p/19q codeleted glioma, the TE 97‐ms PRESS sequence was not designed for optimal detection of cystathionine. The published cystathionine measurements by TE 97‐ms PRESS were very different between the studies in terms of sensitivity and specificity with respect to 1p/19q status [8, 13]. Given the high significance of IDH mutation and 1p/19q codeletion in the clinical management of gliomas [14], it would be valuable to examine the performance of the 2HG‐tailored long‐TE PRESS sequence for evaluating cystathionine and 2HG in a cohort of glioma patients and subsequently 1p/19q and IDH status.

Here, we report TE 97‐ms PRESS measurements of cystathionine and 2HG and their analyses with respect to the 1p/19q and IDH mutational statuses in glioma patients. Group comparison of metabolite estimates is presented together with receiver operating characteristic (ROC) analyses of the MRS measures with respect to the genetic alterations. In addition, cystathionine‐optimized TE 113‐ms PRESS is introduced with data from two glioma patients, including the first MRS imaging of cystathionine in a glioma patient.

## 2 Methods

### 2.1 Study Subject Enrollment

Thirty‐eight patients with biopsy‐proven gliomas were enrolled in the study. The patients had an MRS exam within 3 months before surgery. There was no chemotherapy and/or radiation treatments between the MRS exam and surgery. Patients did not have treatment for 3 months prior to the MRS exam. The enrollment included 18 male and 20 female subjects, with an age range of 25–78 years at the time of MRS exams (mean 44 ± 13 years). The tumors included 29 IDH mutant and nine wildtype gliomas. Of the 29 IDH‐mutant tumors, 15 were 1p/19q codeleted and 14 noncodeleted. IDH‐wildtype gliomas were all 1p/19q noncodeleted. Four healthy adult volunteers (age 26–38 years) were enrolled for control MRS. The brain tumor MR protocols were approved by local Institutional Review Boards. Written informed consent was obtained from each patient prior to the MR scan.

### 2.2 Density‐Matrix Simulation

Computer simulation was performed to determine optimal inter‐radio frequency (RF) pulse timings of PRESS for cystathionine detection at 3 T. The time evolution of the density operator during PRESS was calculated by solving the Liouville‐von Neumann equation for the Hamiltonian that included Zeeman, chemical shift, scalar coupling terms, and slice‐selective RF and gradient pulses, using a product operator‐based transformation matrix algorithm, as described in a previous study [15]. The experimental slice‐selective RF pulses included a 9.8‐ms 90° pulse (bandwidth 4.2 kHz) and a 13.2‐ms 180° RF pulse (bandwidth 1.3 kHz) at an RF field intensity (B_1_) of 13.5 μT (“sharp” pulses in Philips scanners [16]). Transformation matrices were created for the 90° and 180° pulses, incorporating the actual slice‐selective RF and gradient pulse waveforms, and used to calculate spectra of cystathionine and aspartate according to a published algorithm (Supporting Information) [4]. The spatial resolution of the slice selection was set at 1% with respect to the slice thickness, namely, 0.01 = sample length/number of pixels/slice thickness, where the sample length, with number of pixels (isochromats) of 200, was twofold greater than the slice thicknesses. The carrier frequency of the slice‐selective RF pulses was set to 2.5 ppm. Spectra were calculated for TE_1_ and TE_2_ between 15 and 105 ms with 1‐ms increments. The amplitudes of cystathionine and aspartate signals, broadened to a singlet full width at half magnitude (FWHM) of 5 Hz, were examined to determine optimal PRESS subecho times for cystathionine detection. Published chemical‐shift and J‐coupling constants were used in the simulation [9, 10]. The simulation was programmed with Matlab (The MathWorks Inc.).

### 2.3 Synthesis of Cystathionine

The synthesis of L‐cystathionine was accomplished by a modification of published procedures [17].

*Synthesis of (4S)‐1,3‐thiazane‐2,4‐dicarboxylic acid hydrochloride [(4S)‐1,3‐TDC·HCl]*: Dichloroacetic acid (25.78 g, 0.200 mol) was added to a solution of L‐methionine (7.46 g, 50.0 mmol) in concentrated hydrochloric acid (50 mL). After refluxing overnight (100°C), the reaction mixture was placed in the ice bath for 8 h. The precipitated (*4S*)‐1,3‐TDC·HCl was filtered and washed with tetrahydrofuran (THF) and dried. Yield: 6.88 g (60%). ^1^H‐NMR (400 MHz, D_2_O): δ 5.08 (1H, s), 3.95 (1H, dd), 3.03 (1H, m), 2.86 (1H, m), 2.52 (1H, m), 1.88 (1H, m). ^13^C NMR (100 MHz, D_2_O): δ 173.31, 170.73, 61.63, 60.53, and 29.61 ppm.

*Synthesis of L‐homocysteine*: (*4S*)‐1,3‐TDC·HCl (6.83 g, 0.030 mol) was dissolved in ethanol (100 mL). The mixture was adjusted to pH 7 using triethylamine. Ethanolic hydroxylamine hydrochloride (60 mL, 0.5 M) was added to the mixture, and the pH was adjusted to seven with triethylamine. The mixture was refluxed at 80°C for 6 h, then allowed to stand overnight at room temperature. The precipitated L‐homocysteine was filtered, washed with methanol, and dried. Yield: 3.65 g (90%). ^1^H NMR (400 MHz, D_2_O): δ 3.78 (1H, dd), 2.69 (2H, m), 2.01 (2H, m). ^13^C NMR (100 MHz, D_2_O): δ 175.5, 55.2, 36.1, and 21.3 ppm.

*Synthesis of L‐cystathionine [(2S,2′R)‐2‐amino‐4‐(2′‐amino‐2′‐carboxyethylsulfanyl)butanoic acid]*: L‐Homocysteine (3.38 g, 0.025 mol) was dissolved in NaOH (20 mL, 5 M) followed by the addition of *R*‐2‐animo‐3‐chloropropanoic acid as a solid. The mixture was stirred at room temperature for 24 h. It was then cooled in an ice bath and titrated with HCl (5 M) to pH ~3, upon which the product L‐cystathionine slowly precipitated. It was filtered and washed with water and ethanol and dried. Yield: 4.78 g (86%). ^1^H‐NMR (400 MHz, 1‐M DCl in D_2_O): δ 4.15 (1H, dd), 4.03 (1H, t), 3.04 (1H, dd), 2.98 (1H, dd), 2.68 (2H, t), 2.59 (2H, m). ^13^C NMR (100 MHz, 1‐M DCl in D_2_O): δ 171.47, 170.36, 52.06, 51.37, 30.49, 29.20, and 26.80 ppm.

### 2.4 MRS Data Acquisition

MR experiments were carried out on Philips whole‐body 3‐T scanners, using a body coil for RF transmission and a 32‐channel phased‐array head coil for reception. Phantom MRS data were obtained from two aqueous solutions at neutral pH and room temperature: one with 1‐mM cystathionine and 5‐mM glycine and the other with aspartate and glycine both at 30 mM. PRESS data were collected at TEs of 97 and 113 ms (TR = 2 s; voxel size = 25 × 25 × 25 mm^3^). The number of signal averages was 1024 and 32 in the cystathionine and aspartate phantom experiments, respectively.

For in vivo MR scans in brain tumor patients, T2‐FLAIR was obtained for tumor identification (TR/TE/TI = 9000/125/2600 ms; FOV = 230 × 200 mm^2^; slice thickness = 4 mm). Single‐voxel localized MRS was acquired from the entire study subjects, using TE 97‐ms PRESS (TE_1_ = 32 ms and TE_2_ = 65 ms). A voxel was positioned within the T2‐FLAIR hyperintense volume, with care taken to avoid cysts and/or necrotic regions. The voxel size ranged from 2.1 to 11.4 mL (mean 6.8 ± 2.0 mL), and the number of water‐suppressed signal averages was set to range from 768 to 128 (mean 213 ± 136) (scan time 26–4.3 min) to achieve similar SNR across the voxel size (Figure S1). The MRS acquisition parameters included 2500‐Hz sweep width, 2048 sampling points, and 2‐s TR. Water‐suppressed time‐domain signals were recorded in multiple blocks, each with 16 RF phase cycled signal averages. Unsuppressed water was acquired at the beginning of each block for multichannel combination and eddy‐current compensation. Water suppression was obtained with a four‐pulse variable flip angle scheme [18]. B_1_ calibration was performed on the shim volume (22–27 mL). Up to second‐order B_0_ shimming was carried out using a vendor‐supplied tool (pencil beam). The carrier frequency of the PRESS RF pulses was set at 2.7 ppm. Frequency drifts were corrected in real time for each excitation using a vendor‐supplied tool (frequency stabilization). In addition, unsuppressed water was acquired with 14‐ms TE and 20‐s TR for use as a reference in metabolite quantification.

TE 113‐ms PRESS data, which was optimized for cystathionine detection (TE_1_ = 42 ms and TE_2_ = 71 ms), was acquired from two glioma patients. In addition to single‐voxel localized TE 97‐ and 113‐ms PRESS acquisitions, 2D chemical‐shift imaging (CSI) of cystathionine was carried out in a patient. The TE 113‐ms PRESS sequence was used to prescribe a 70 × 70‐mm^2^ volume of interest (VOI) within a 90 × 90‐mm^2^ field of view (FOV). The CSI data were recorded with 10 × 10‐mm^2^ resolution and 4 averages per k‐space point (slice thickness = 15 mm; TR = 1.5 s; scan time = 8.1 min).

Single‐voxel localized TE 97‐ and 113‐ms PRESS data were acquired from the white‐matter regions of four healthy subjects. Unsuppressed water was recorded with 20‐s TR and 14‐ms TE. T1‐weighted water images were used for voxel positioning.

### 2.5 MRS Data Processing

The single‐voxel multiblock MRS data were corrected for frequency drifts prior to final signal averaging. Following apodization with a 1‐Hz exponential function, spectral fitting was performed with LCModel software (Version 6.3‐1L) [19]. The basis set included calculated spectra of 22 metabolites, which included 2HG, cystathionine, aspartate, glutamate, glutamine, γ‐aminobutyric acid, myo‐inositol, glycine, lactate, citrate, glutathione, ethanolamine, phosphoethanolamine, scyllo‐inositol, taurine, glucose, N‐acetylaspartate (NAA), N‐acetylaspartylglutamate (NAAG), glycerophosphocholine, phosphocholine, creatine, and phosphocreatine. The metabolites' basis spectra were numerically calculated with an in‐house density‐matrix simulation tool, as described above. Spectral apodization was not applied in the basis set preparation. The basis set also included two in‐house lipid bases and the LCModel default bases of the lipid 1.3 ppm resonances (Lip13a and Lip13b). In one of the customized lipid bases, the strength of the lipid resonance at the 2HG 2.25‐ppm resonance was set to be 60% of the lipid 0.9‐ppm signal strength, similarly to a prior study [20]. LCModel default lipid bases, Lip20 and Lip09, and macromolecular bases were excluded in the spectral fitting. LCModel fitting was conducted between 0.2 and 4.0 ppm. The percentage standard deviations of metabolite signal estimates were returned by LCModel. The LCModel estimates of metabolite signals were normalized to the TE 14‐ms and TR 20‐s water signal for individual subjects, and subsequently, the millimolar concentrations of metabolites were calculated by scaling the mean metabolite‐to‐water signal ratios for total creatine (tCr) in healthy‐brain white‐matter regions at 6 mM (Figure S2) [21], assuming that the T1 and T2 relaxation effects are identical between metabolites and between tumor and healthy brain.

The 2D CSI k‐space data were twofold zero‐filled and Fourier transformed along the k‐space directions using the scanner built‐in algorithm. Excluding the data in the outer volume of the 70 × 70‐mm^2^ VOI, where signals were suboptimal due to the chemical‐shift associated VOI displacements, the time‐domain data in the 50 × 50‐mm^2^ volume were included in the subsequent data analysis. The LCModel fitting was performed on spectra apodized with a 1‐Hz exponential function. The metabolite concentrations were estimated with reference to tCr in the normal‐appearing white‐matter region at 6 mM [22].

Group comparison was performed with an unpaired *t*‐test. The sensitivity and specificity of MRS measures with respect to IDH and 1p/19q mutational statuses were evaluated with ROC curve analysis. Statistical significance was declared at *p* < 0.05. Data are presented as mean ± standard deviation.

## 3 Results

**FIGURE 1:**
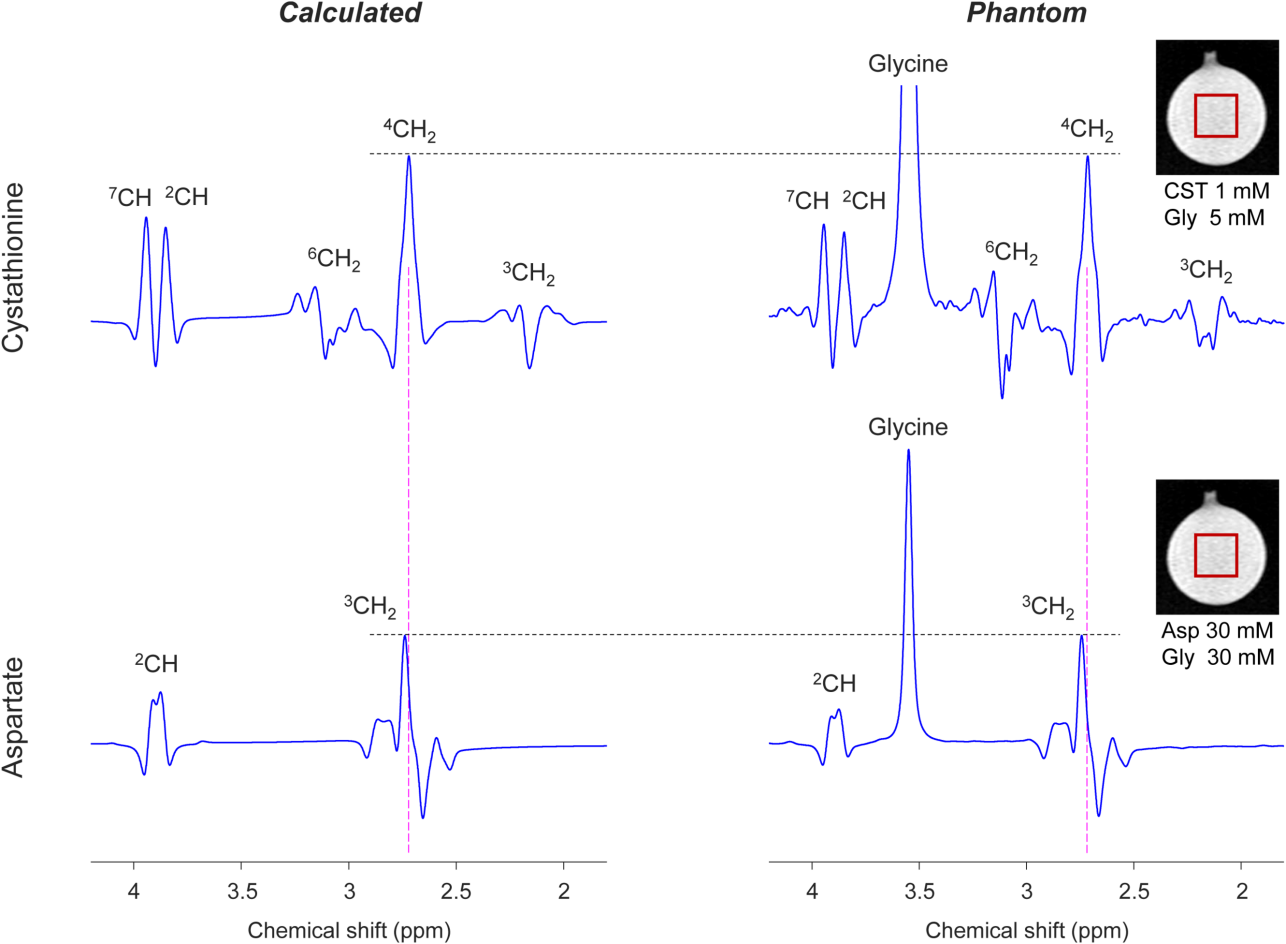
Phantom spectra of cystathionine (upper panel) and aspartate (lower panel), obtained with TE 97‐ms PRESS (TE_1_ = 32 ms and TE_2_ = 65 ms), are presented in the right column, and calculated TE 97‐ms PRESS spectra of the metabolites at equal concentrations are shown in the left column. After broadening to a singlet FWHM of 5 Hz, spectra were scaled to match the amplitudes of cystathionine and aspartate peaks around 2.72 ppm between calculated and phantom spectra, as indicated by horizontal dashed lines. Vertical dashed lines are drawn at 2.72 ppm.

In phantom and calculated TE 97‐ms PRESS spectra of cystathionine and aspartate (Figure 1), the C4‐proton spins of cystathionine give rise to the highest peak at 2.72 ppm among its five resonances and the peak is overlapped by the largest signal of aspartate. The second largest signal of cystathionine arose from the C2‐proton and C7‐proton spins, and the resulting dual peaks around 3.9 ppm coincided with the C2‐proton signal of aspartate. Other signals of cystathionine were relatively small.

**FIGURE 2:**
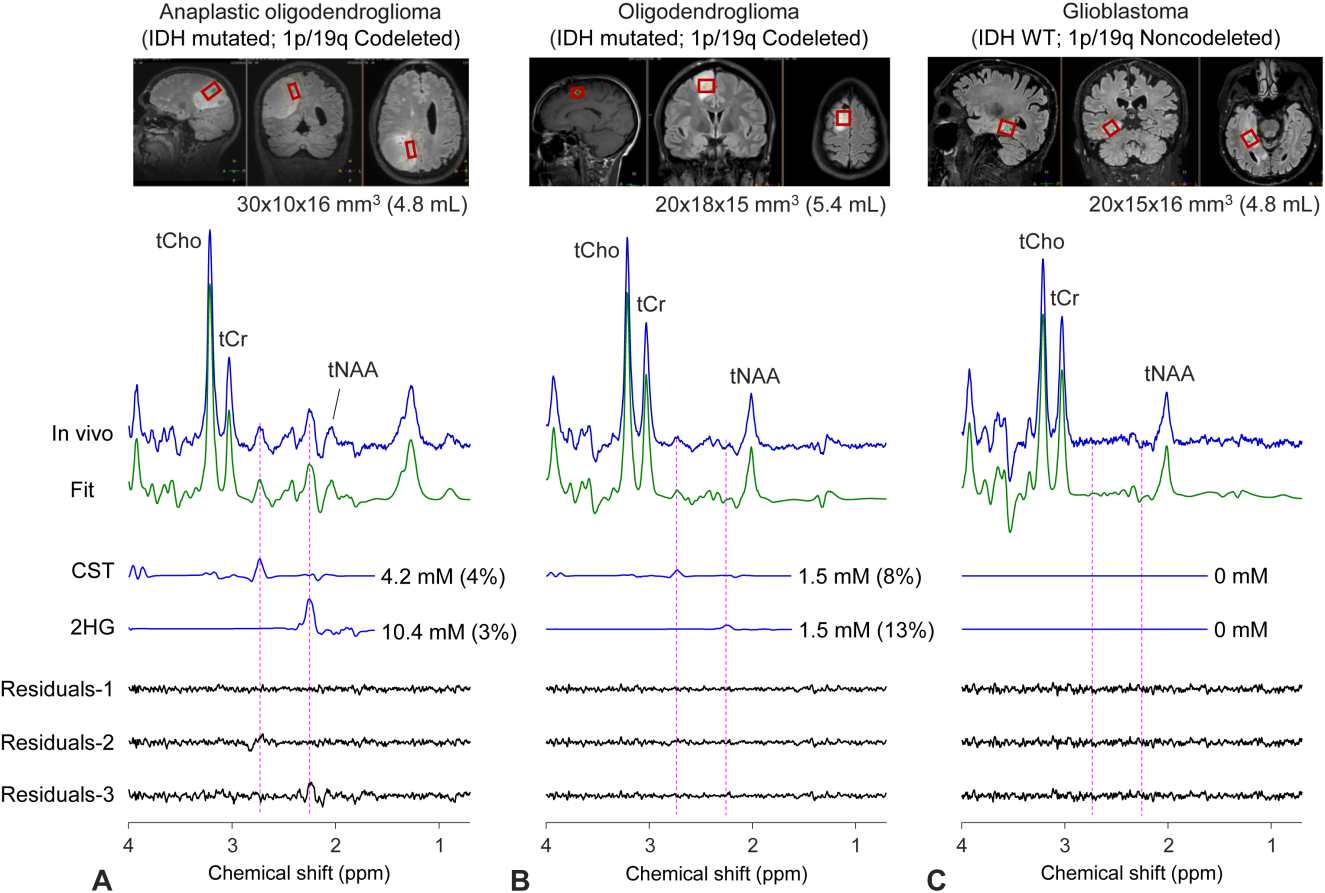
Representative TE 97‐ms PRESS spectra from three glioma patients are presented together with the voxel positioning on T2‐FLAIR images. LCModel‐returned cystathionine (CST) and 2HG signals are shown with the millimolar concentration estimates and percentage standard deviation values in brackets. Three types of residuals are presented for each patient data: Residuals‐1 represents the residuals from spectral fitting with the full basis set having both CST and 2HG, Residuals‐2 indicates the residuals from spectral fitting with a basis set excluding CST, and Residuals‐3 shows the residuals from spectral fitting with a basis set excluding 2HG. Spectra are scaled with respect to the water signals from individual voxels. The number of signal averages was 192 for each spectrum (TR = 2 s; scan duration = 6.4 min). Vertical lines are drawn at the CST and 2HG signal locations (2.72 and 2.25 ppm).

Figure 2 presents representative in vivo spectra from three glioma patients. A 40‐year‐old male patient with a right parietal mass had an MRS exam 1 day before surgery. In the spectrum from a noncubic voxel positioned in the more solid portion of the tumor, large signals were readily identifiable at 2.72 and 2.25 ppm (Figure 2A). Spectral analysis resulted in concentrations of cystathionine and 2HG at 4.2 and 10.4 mM, respectively, when quantified with reference to healthy‐brain white‐matter tCr at 6 mM (Figure S2). Tumor tissue analysis showed the lesion was an IDH1‐mutated, 1p/19q codeleted anaplastic oligodendroglioma, in line with MRS observation of elevated cystathionine and 2HG. Spectral fitting was additionally performed excluding cystathionine and 2HG in the basis set. Compared to the residuals from the fitting with the full basis set, the residuals from the fitting without cystathionine or 2HG in the basis set resulted in large unfit residues around 2.72 or 2.25 ppm, respectively, which indicated that abundant signals of cystathionine and 2HG were present in the spectrum. Next, MRS was acquired from a recurrent tumor of an oligodendroglioma patient 2 months before the second surgery (Figure 2B). Signals at 2.72 and 2.25 ppm were relatively small, resulting in estimates of cystathionine and 2HG both at 1.5 mM. Marginal differences were seen between residuals from the three types of LCModel fitting. IDH mutation and codeleted 1p/19q were observed in tissue analysis. Lastly, MRS was undertaken 15 days before surgery in a 78‐year‐old male patient, in whom biopsy analysis showed that the lesion had wildtype IDH and intact 1p/19q. Null concentrations of cystathionine and 2HG resulted (Figure 2C). There was no difference seen between residuals from the three types of spectral fitting.

For the three spectra in Figure 2, which were apodized with a 1‐Hz exponential function, the total choline (tCho) singlet FWHM was, from left to right, 6.7, 6.7, and 7.0 Hz, and the tCho signal‐to‐noise ratio (SNR) was 119, 150, and 83, when SNR was calculated as a ratio of LCModel‐returned tCho peak amplitude to the standard deviation of the residuals between 0.2 and 4.0 ppm. For the spectra from the entire 38 patients of the present study (Figure S3), the tCho FWHM and SNR were 6.2 ± 1.2 Hz (range 4.6–9.4 Hz) and 110 ± 42 (range 34–204), respectively. The tCho concentration ranged from 1.5 to 12.5 mM (mean 3.3 ± 1.8 mM). The water FWHM, without apodization, was 4.1 ± 1.2 Hz (range 2.1–8.1 Hz).

**FIGURE 3:**
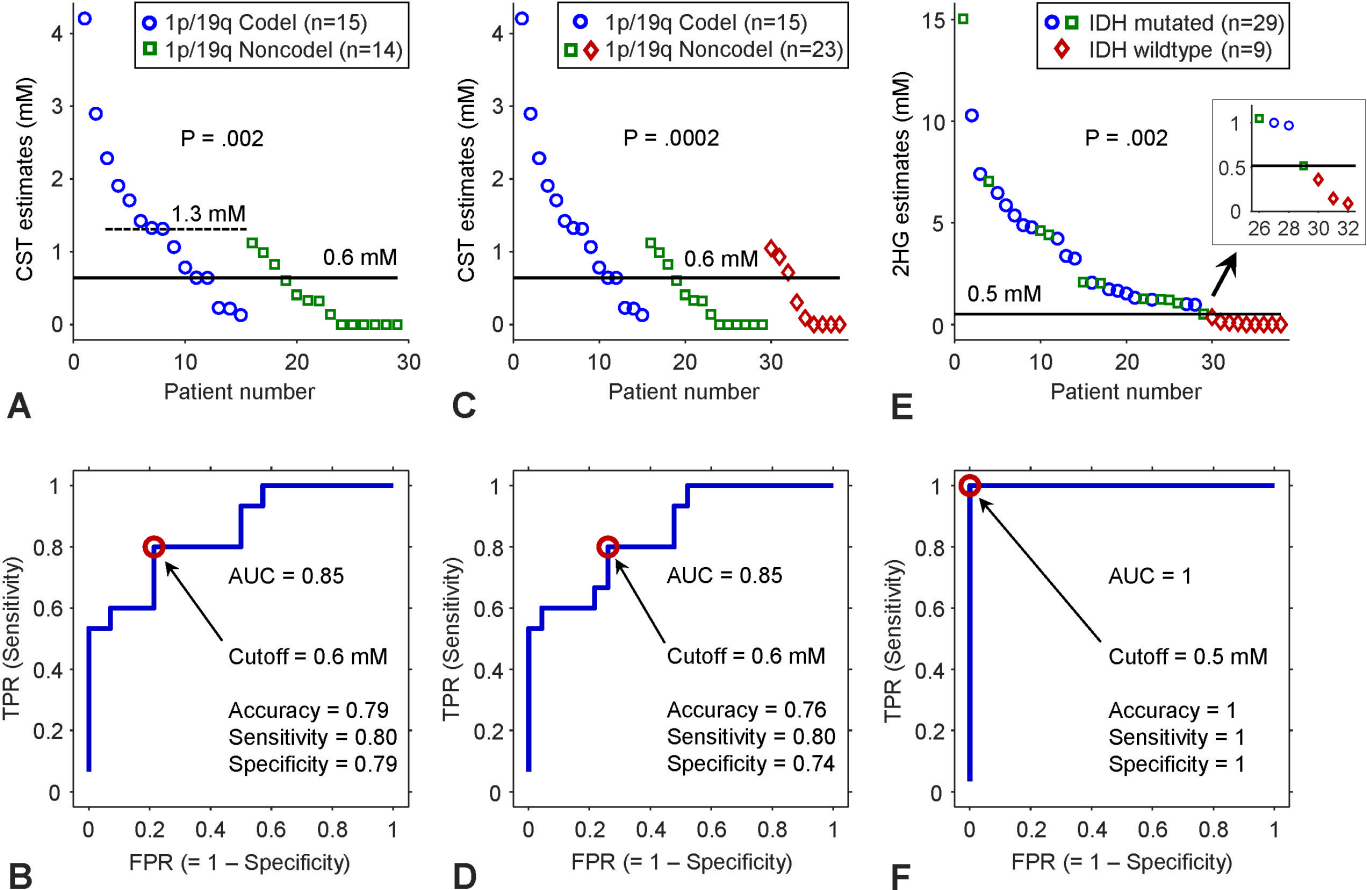
(A, B) Cystathionine (CST) estimates from 29 glioma patients are presented in the upper panel (15 1p/19q codeleted and 14 noncodeleted gliomas). The lower panel shows a plot of true positive rate (TPR) vs. false positive rate (FPR) from a receiver operating characteristic (ROC) analysis of the CST estimates with respect to 1p/19q status. The 29 tumors were all IDH mutated. (C, D) CST estimates from 38 glioma patients are presented in upper panel (15 1p/19q codeleted and 23 noncodeleted gliomas), and the lower panel shows a plot of TPR vs. FPR for the CST estimates with respect to 1p/19q status. The nine tumors shown by red diamonds were IDH wildtype. (E, F) 2HG estimates from the 38 glioma patients are presented in the upper panel (29 IDH mutated and nine wildtype gliomas), and ROC analysis of the 2HG estimates with respect to IDH mutational status is shown in the lower panel. For each of the upper panel figures, a *p*‐value represents the statistical significance of CST difference (A and C) or 2HG difference (E) between the two groups indicated in the legend. For each of the (A, B), (C, D), and (E, F) figure pairs, a horizontal solid line in the upper panel figure indicates a CST or 2HG cutoff that corresponds to a red circle on the ROC curve, from which the ROC curve was closest to the top‐left corner (FPR = 0 and TPR = 1). Each lower panel figure shows the area under the ROC curve (AUC) as well as accuracy, sensitivity, and specificity with respect to 1p/19q or IDH status that were calculated with the cutoff value. In figure (A), a horizontal dashed line is drawn at 1.3 mM, above which the eight tumors were all 1p/19q codeleted. Inset in figure (E) shows the 2HG estimates in the proximity of the cutoff value (0.5 mM).

For the 15 1p/19q codeleted and 14 noncodeleted tumors, TE 97‐ms PRESS resulted in cystathionine levels of 1.4 ± 1.1 mM (range 0.1–4.2 mM) and 0.3 ± 0.4 mM (range 0–1.1 mM), respectively (Figure 3A). The cystathionine estimate difference between the 1p/19q codeleted and noncodeleted tumor groups was statistically significant (*p* = 0.002). Tumors with cystathionine estimates higher than 1.3 mM were all 1p/19q codeleted. The aspartate estimates were much lower than the cystathionine estimates in 1p/19q codeleted gliomas and not significantly different between the 1p/19q codeleted and noncodeleted tumors (0.5 ± 0.4 vs. 0.3 ± 0.3 mM; *p* = 0.1). ROC analysis was performed with the cystathionine estimates from the 29 patients (Figure 3B). When the true positive rate (TPR) was plotted versus the false positive rate (FPR), the area under the ROC curve (AUC) was calculated to be 0.85. A cystathionine cutoff corresponding to the point closest to the top‐left corner of the axes (i.e., FPR = 0 and TPR = 1) was obtained as 0.6 mM. Accuracy, sensitivity, and specificity with respect to 1p/19q status were calculated as 0.79, 0.80, and 0.79, respectively. Figure 3C shows cystathionine estimates in all 38 tumors, including the nine IDH‐wildtype 1p/19q noncodeleted gliomas. The statistical significance of the cystathionine estimate difference between 15 1p/19q codeleted and 23 noncodeleted tumors was much stronger (1.4 ± 1.1 vs. 0.4 ± 0.4 mM; *p* = 0.0002), while an ROC analysis of the cystathionine estimates with respect to 1p/19q status resulted in the same sensitivity (0.80) and a slightly lower specificity (0.74), with an unchanged cystathionine cutoff value (0.6 mM) (Figure 3D). The aspartate estimates were about the same between the 1p/19q codeleted and noncodeleted tumors (0.5 ± 0.4 vs. 0.4 ± 0.5 mM; *p* = 0.7). Group comparison and ROC analysis were also performed with 2HG estimates from the 38 patients with respect to IDH mutational status (Figure 3E,F). The 2HG estimate was much higher in the 29 IDH‐mutant tumors than in the nine IDH‐wildtype tumors (3.7 ± 3.3 vs. 0.1 ± 0.1; *p* = 0.002). The statistical significance of the 2HG estimate difference between IDH‐mutant and IDH‐wildtype tumors was about the same as that of the cystathionine estimate difference between the 15 codeleted and 14 noncodeleted tumors, but the ROC curve was very different. A 2HG cutoff value of 0.5 mM resulted in a complete distinction between IDH mutation and IDH‐wildtype tumors; the area under the ROC curve was unity, and the accuracy, sensitivity, and specificity of 2HG estimation with respect to IDH status were all unity. The GABA estimates were much lower than the 2HG estimates in IDH‐mutant gliomas and very similar between the IDH‐mutant and IDH‐wildtype tumors (0.4 ± 0.4 vs. 0.4 ± 0.3 mM; *p* = 0.7).

**FIGURE 4:**
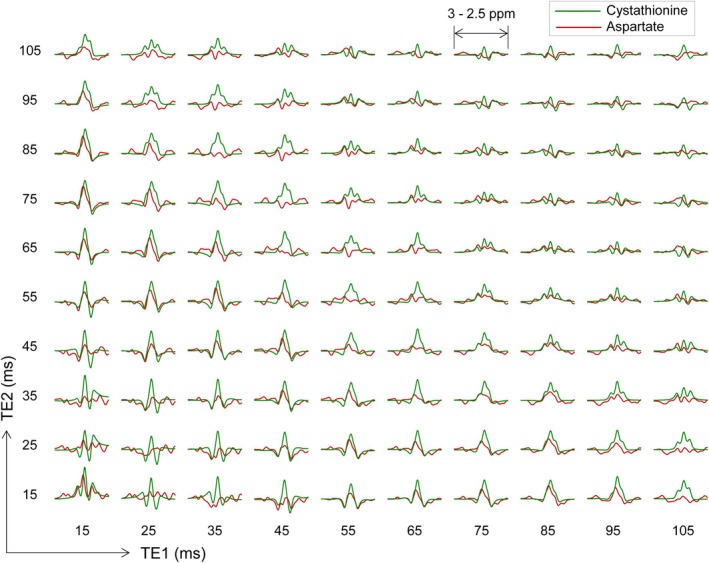
Numerically calculated signals of cystathionine (green) and aspartate (red) are plotted versus PRESS subecho times TE_1_ and TE_2_ (90—TE_1_/2—180—TE_1_/2—TE_2_/2—180—TE_2_/2—Acquisition). For each (TE_1_ and TE_2_) set, the C4‐proton signal of cystathionine and the C3‐proton signal of aspartate, broadened to a singlet FWHM of 5 Hz, are displayed on top of each other between 2.5 and 3 ppm. The signal strength was adjusted with a T2 of 180 ms for both cystathionine and aspartate.

Figure 4 presents calculated cystathionine C4‐proton signals vs. PRESS subecho times, TE_1_ and TE_2_. When the signal strengths were scaled with a T_2_ of 180 ms, which is a published brain glutamate T_2_ [23], the cystathionine C4‐proton and aspartate C3‐proton multiplets were the largest at a TE of 30 ms, with extensive overlaps between the signals. At subecho times for TE_1_ + TE_2_ of 95–100 ms, the cystathionine and aspartate multiplets were both simplified to a single‐peak pattern with relatively high amplitudes. For TE_1_ = 40–45 ms and TE_2_ = 70–75 ms, the aspartate signal strength at ~2.72 ppm was substantially decreased compared to those at TEs of 95–100 ms, while the cystathionine signal showed a slight reduction with the TE lengthening.

**FIGURE 5:**
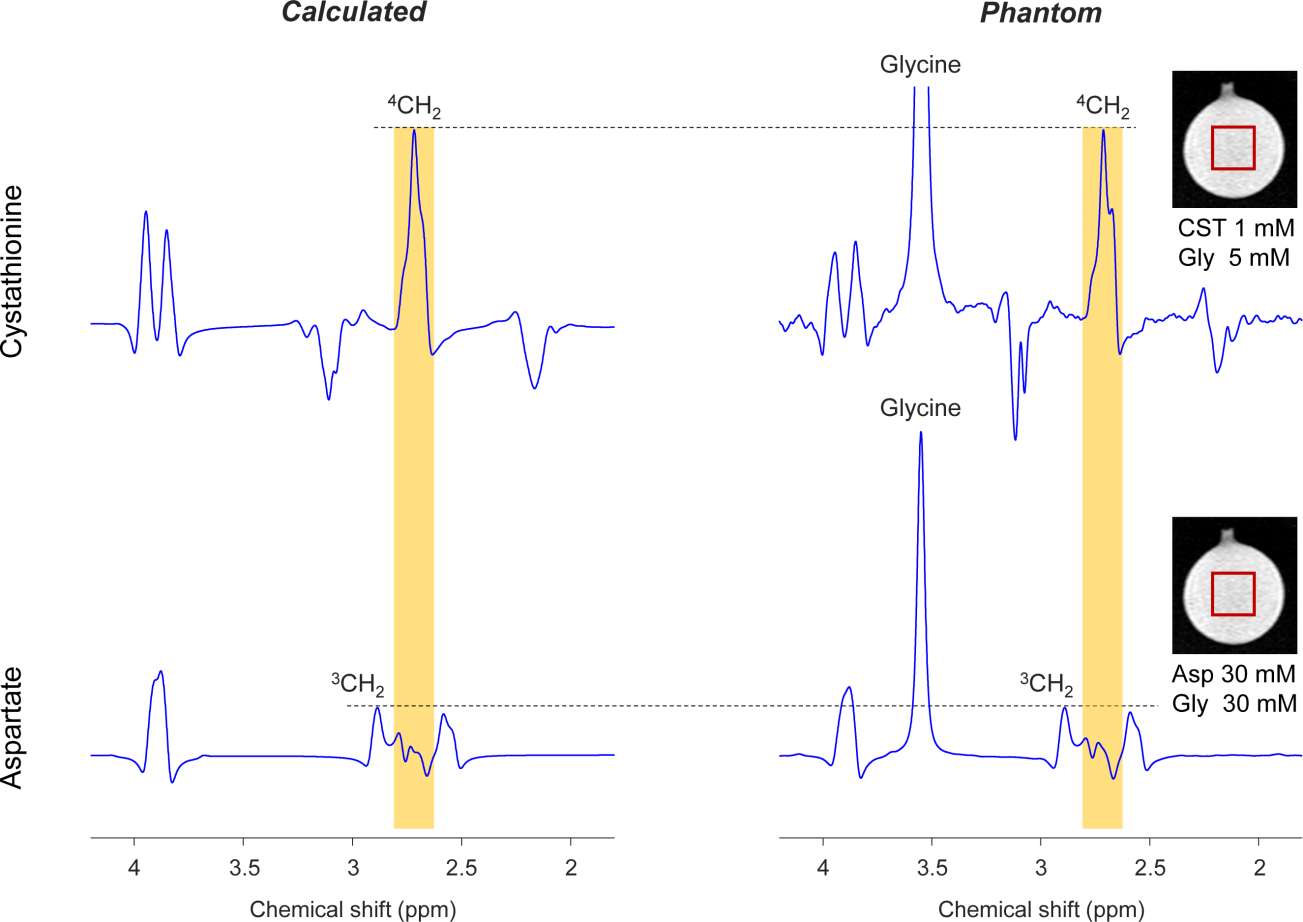
Phantom spectra of cystathionine (upper panel) and aspartate (lower panel), obtained with TE 113‐ms PRESS (TE_1_ = 42 ms and TE_2_ = 71 ms), are presented in the right column, and calculated TE 113‐ms PRESS spectra of the metabolites at equal concentrations are shown in the left column. After broadening to singlet FWHM of 5 Hz, spectra were scaled to match the amplitudes of cystathionine and aspartate peaks around 2.72 ppm between calculated and phantom spectra, as indicated by horizontal dashed lines. A spectral region of 2.62–2.82 ppm is highlighted in yellow.

Simulations with 1‐ms increments of PRESS subecho times suggested that a subecho time set of TE_1_ and TE_2_ at 42 and 71 may provide detection of the cystathionine 2.72‐ppm resonance with minimal interference from the overlapping aspartate resonances. Phantom and calculated spectra of cystathionine and aspartate at this PRESS TE of 113 ms are presented in Figure 5. For a spectral range of 2.62–2.82 ppm, the aspartate C3‐proton signal amplitude was about 10% with respect to the cystathionine 2.72‐ppm peak amplitude. TE 113‐ms PRESS signals of cystathionine and aspartate other than those between 2.5 and 3.0 ppm were not very different from their TE 97‐ms PRESS signals in Figure 1.

**FIGURE 6:**
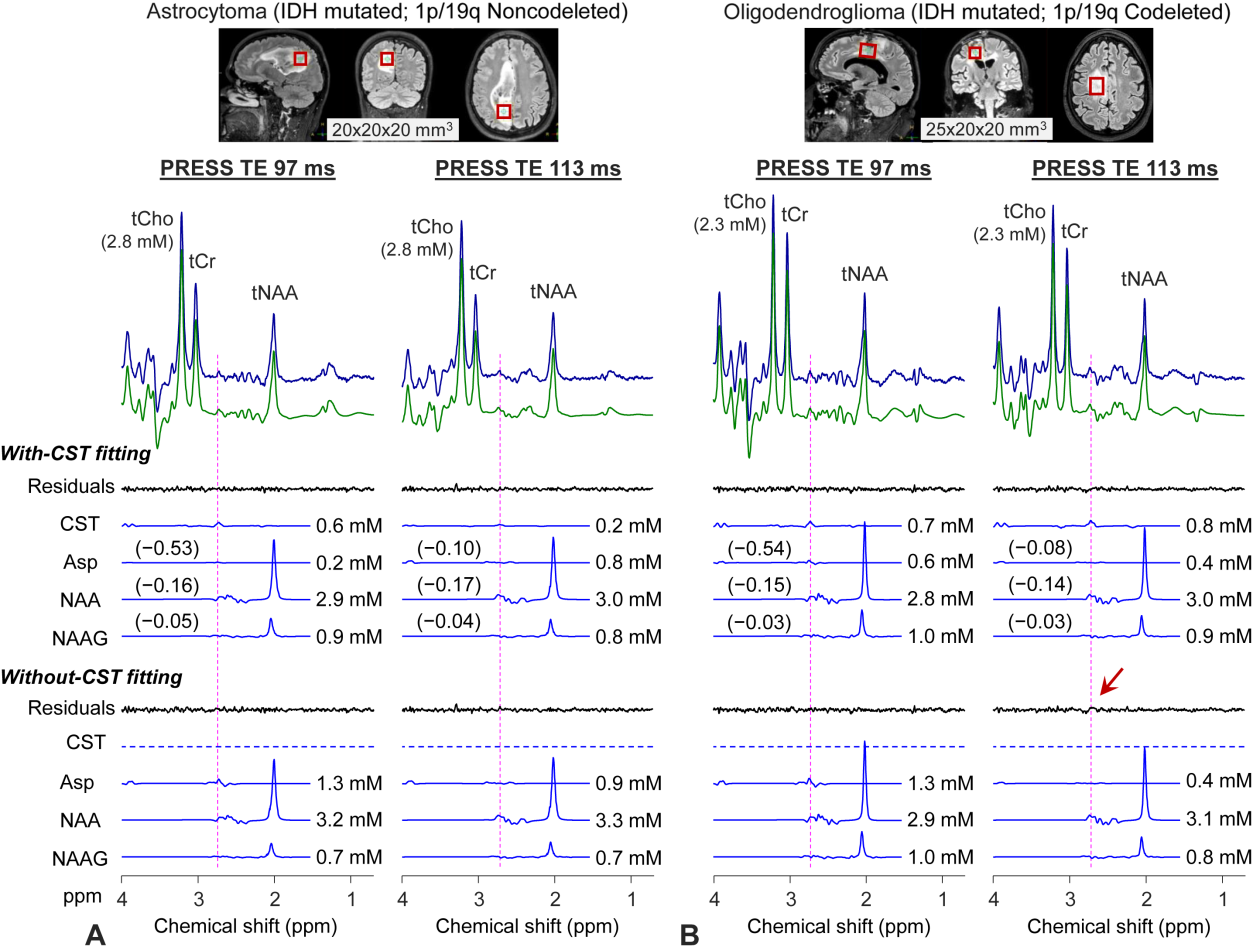
In vivo TE 97‐ and 113‐ms PRESS spectra from (A) an astrocytoma (1p/19q noncodeleted) and (B) an oligodendroglioma (1p/19q codeleted) patients are presented together with spectral fitting outputs and voxel positionings on T2‐FLAIR images. LCModel‐returned residuals and signals of cystathionine (CST), aspartate (Asp), NAA, and NAAG are presented for two types of fittings: one fitting with the full basis set (With‐CST fitting) and the other fitting without CST in the basis set (without‐CST fitting). The metabolite concentration estimates from the fittings are displayed, wherein the mM values of TE 113‐ms PRESS were corrected with a T2 of 280 ms. LCModel‐returned correlation coefficients of Asp, NAA, and NAAG with respect to CST are presented for the With‐CST fitting. The number of signal averages was 128 for each spectrum (TR = 2 s). Vertical lines are drawn at 2.72 ppm. A red arrow indicates the unfit residuals in the without‐CST fitting.

The cystathionine‐optimized TE 113‐ms PRESS sequence was tested in two glioma patients, together with the TE 97‐ms PRESS. In a patient with a 1p/19q noncodeleted astrocytoma (Figure 6A), cystathionine and aspartate estimations using TE 97‐ms PRESS were 0.6 and 0.2 mM, respectively. In contrast, the TE 113‐ms PRESS method resulted in 0.2‐mM cystathionine and 0.8‐mM aspartate when corrected for T2 relaxation effects using a published tCho T2 value in brain tumors (280 ms) [24]. The LCModel‐returned correlation coefficient between cystathionine and aspartate was smaller in TE 113‐ms PRESS than in TE 97‐ms PRESS (−0.10 vs. −0.53), indicating that the two metabolites can be estimated with smaller dependence on each other using the TE 113‐ms PRESS. The correlations of cystathionine with NAA and NAAG were relatively small in both PRESS methods. Aspartate estimation rose by sixfold when fitting the TE 97‐ms PRESS data without cystathionine (from 0.2 to 1.3 mM), while the effect of without‐cystathionine fitting on aspartate estimation was minimal in TE 113‐ms PRESS (0.8 vs. 0.9 mM). The effects of without‐cystathionine fitting on NAA and NAAG estimations were relatively small in both TE 97‐ and 113‐ms PRESS data. For an IDH‐mutant oligodendroglioma with 1p/19q codeletion (Figure 6B), estimates of cystathionine, aspartate, NAA, and NAAG were not very different between TE 97‐ and 113‐ms PRESS data. The LCModel‐returned correlation coefficient between cystathionine and aspartate was sevenfold smaller in TE 113‐ms PRESS than in TE 97‐ms PRESS (−0.08 vs. −0.54). In line with these correlation coefficient values, a change in aspartate estimation by without‐cystathionine fitting was minimal in the TE 113‐ms data compared to the TE 97‐ms data (i.e., from 0.4 to 0.4 mM vs. from 0.6 to 1.3 mM). For the TE 113‐ms PRESS data, a difference in residuals between with‐ and without‐cystathionine fittings was noticeable.

**FIGURE 7:**
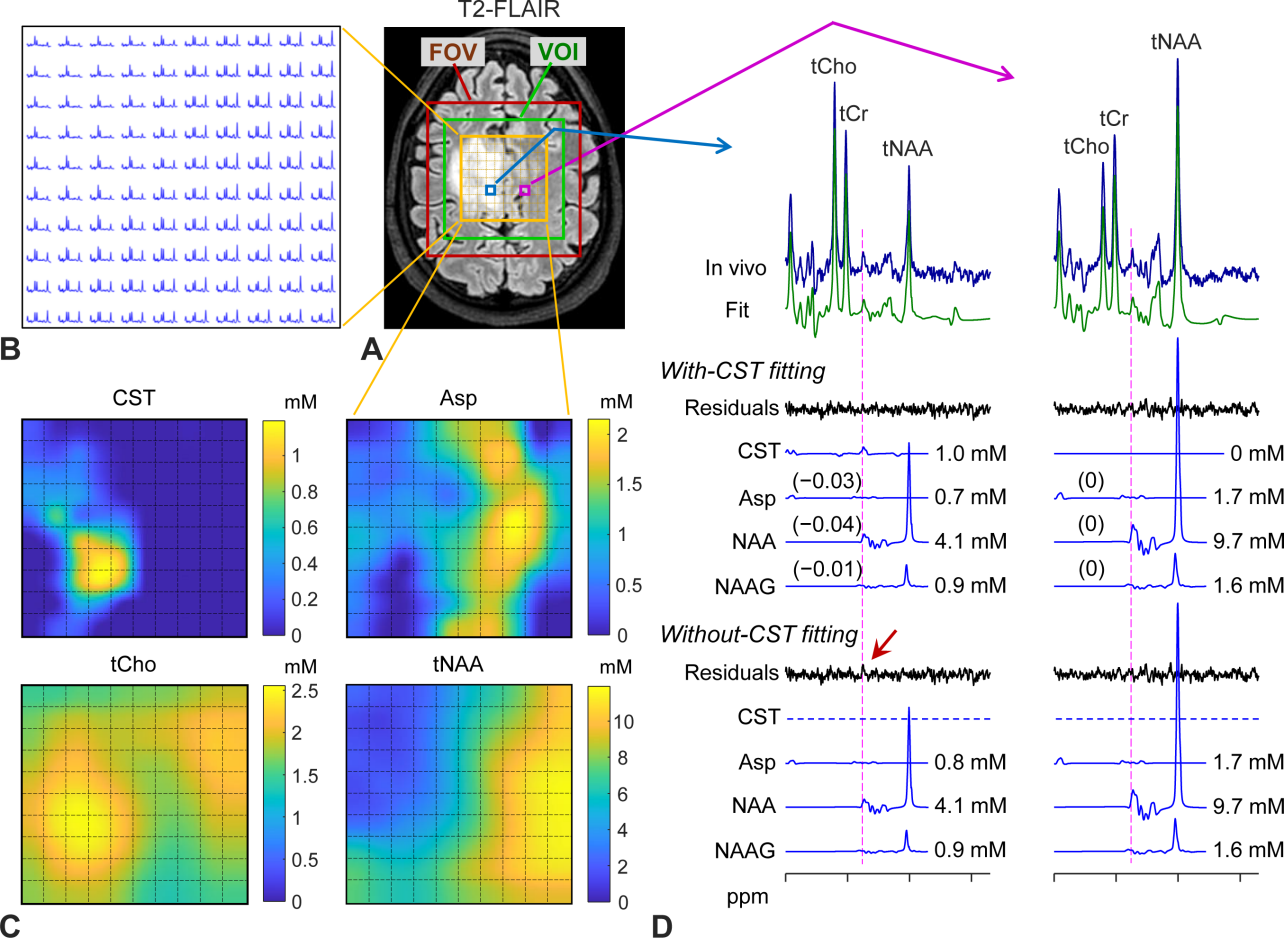
In vivo 2D chemical‐shift imaging in an oligodendroglioma patient (1p/19q codeleted). (A, B) 10 × 10 spectra are shown for a 50 × 50‐mm^2^ volume (indicated by a yellow box on a T2‐FLAIR axial image) within a 70 × 70‐mm^2^ volume of interest (VOI) (green box). The spectra, apodized with a 1‐Hz exponential function, are displayed for 1.7–4 ppm. The VOI was prescribed with TE 113‐ms PRESS in the imaging scan (FOV = 90 × 90 mm^2^; slice thickness = 15 mm; 4 averages per k‐space point). The k‐space data, recorded for 10 × 10‐mm^2^ resolution, were zero filled by twofold along the two directions prior to the Fourier transformation. (C) The concentrations of cystathionine (CST), aspartate (Asp), tCho, and tCr, estimated with reference to tCr in the white‐matter region at 6 mM, are color‐mapped for the 50 × 50‐mm^2^ volume. Each grid within the maps represents 5 × 5 mm^2^. (D) Two representative spectra (one from T2‐FLAIR hyperintense volume and the other from normal‐appearing brain) are shown together with the outputs from with‐CST fitting and without‐CST fitting in a similar fashion to Figure 6. Vertical lines are drawn at 2.72 ppm. A red arrow indicates the unfit residuals in the without‐CST fitting.

The TE 113‐ms PRESS scheme was tested for multivoxel imaging of cystathionine in a patient. The subject with oligodendroglioma, whose single‐voxel MRS data are shown in Figure 6B, was scanned with a PRESS prescription of a 70 × 70‐mm^2^ VOI that included the T2‐hyperintense volume and contralateral brain (Figure 7A). A 2D stack of spectra (Figure 7B), where each spectrum corresponds to 5 × 5 mm^2^, showed well‐defined metabolite peaks on flat backgrounds, which was due to the attenuation of macromolecular signals during the 113‐ms TE. The pattern of tCho, tCr, and total NAA (tNAA) signals was clearly different between the tumor and contralateral normal‐appearing brain regions. A map of cystathionine estimates (Figure 7C) showed that cystathionine was concentrated at the posterior region of the T2‐FLAIR hyperintense volume and was undetectable in the contralateral brain. Aspartate level was overall much lower in the tumor than in the contralateral region. tCho was high in the high‐cystathionine region. Figure 7D shows a spectrum from the high‐cystathionine region and a spectrum from the contralateral brain. While there were negligible differences in estimates of aspartate, NAA, and NAAG between with‐ and without‐cystathionine fittings, a difference in residuals between the fittings was noticeable at 2.72 ppm.

## 4 Discussion

We report MRS measurements of cystathionine and 2HG in 38 glioma patients and analyses of the measures with respect to the 1p/19q and IDH statuses of the tumors. The TE 97‐ms PRESS data showed significantly higher cystathionine concentration in 1p/19q codeleted tumors compared with 1p/19q noncodeleted tumors, confirming the elevation of cystathionine in 1p/19q codeleted gliomas as reported in prior studies [8, 13, 25]. The presence of elevated cystathionine on MRS is evident when large residuals are returned at cystathionine signal locations in spectral fitting excluding cystathionine (Figure 2A), in which case MRS detection of cystathionine can be a strong indicator of 1p/19q codeletion. However, in data from tumors with modest levels of cystathionine, with‐ and without‐cystathionine fittings may not show noticeable differences in residuals. In our TE 97‐ms PRESS data, the sensitivity and specificity of cystathionine measures with respect to 1p/19q status were much lower (80% and 79%, respectively) compared to the 2HG analysis, which showed complete distinction between IDH‐mutant and IDH‐wildtype tumors (sensitivity/specificity = 100%/100%) with a cutoff value of 0.5 mM (Figure 3). In practice, the 2HG signal corresponding to 0.5–1 mM may not be clearly discernible in the spectrum, and the low 2HG estimations can be deemed 2HG positive only when the data quality is very high.

The sensitivity and specificity of cystathionine measures to 1p/19q status were calculated with a cutoff value that corresponded to the point on the ROC curve closest to the perfect differentiation point, i.e., (FPR, TPR) = (0, 1). Alternatively, a cutoff value is often determined based on the maximum Youden index [26], that is, the maximum vertical distance from the point on the ROC curve to the 45° diagonal line connecting the (0, 0) and (1) points. The resulting cutoff value corresponds to the point on the ROC curve farthest from zero differentiation. For the cystathionine estimates in 29 patients (Figure 3A,B), the cutoff values from the two methods were identical, implying that the 0.6‐mM cutoff concentration may provide well‐balanced sensitivity and specificity. Application of the cutoff value in individual patients would suffer non‐negligible false positive/negative cases. As low estimates of cystathionine (e.g., < 0.5 mM) may not have meaningful differences between them, one may focus on high‐cystathionine cases for identifying 1p/19q codeleted gliomas. For the 29 patients, the eight cases with cystathionine higher than 1.3 mM were all 1p/19q codeleted. This suggests that the TE 97‐ms PRESS approach of the present study may be applicable for predicting 1p/19q codeletion based on cystathionine estimation higher than 1.3 mM.

Our cystathionine MRS result of low sensitivity and specificity to 1p/19q status, obtained with TE 97‐ms PRESS, is similar to those of a prior study using TE 97‐ms PRESS (sensitivity/specificity = 92%/61%) [8], but overall higher compared with another TE 97‐ms PRESS cystathionine study (sensitivity/specificity = 44.4%/52.5%) [13]. This Zhou et al. study reported higher sensitivity and specificity from TE 45‐ms PRESS measurements of cystathionine (sensitivity/specificity = 66.7%/73.7%). There are large variations in MRS sensitivity and specificity to 1p/19q status between this study and prior studies. Since mass spectrometry measures of cystathionine in 1p/19q codeleted tumor tissues were not distinctly higher than those in 1p/19q noncodeleted tumors [25], as opposed to 2HG whose biopsy measures differed by 2–3 orders of magnitude between IDH‐mutant and IDH‐wildtype tumors [3], care should be taken when interpreting intermediate cystathionine estimates to predict the 1p/19q status in individual patients.

Our cystathionine concentration estimates in IDH‐mutant 1p/19q codeleted gliomas are somewhat lower compared with those of prior Branzoli et al. studies (1.4 vs. ~2.3 mM) [8, 9]. The discrepancy may be because of differences in data acquisition and analysis methods as well as assumptions used in calculating the concentrations. The cystathionine estimates of the present study may be comparable with healthy‐brain white‐matter 6‐mM tCr, with the assumption of identical T1 and T2 between cystathionine and tCr. In addition, significant levels of cystathionine were observed in three out of nine IDH‐wildtype gliomas in the current study (Figure 3C). These cystathionine estimates may not be entirely due to the limited ability of the MRS method used given that (1) significant levels of cystathionine were measured in IDH‐wildtype glioma tissues by mass spectrometry in a prior study [25], (2) a high level of cystathionine (4.5 mM) was detected by MRS in one of two IDH‐wildtype gliomas in a prior study [25], and (3) 1p/19q codeletion was observed in one of eight IDH‐wildtype glioblastomas in a prior pathological study [27]. It follows that cystathionine could be elevated in a subset of IDH‐wildtype gliomas and thus noninvasive identification of oligodendroglioma, which is defined as an IDH‐mutant 1p/19q‐codeleted glioma [14], may necessitate MRS of both 2HG and cystathionine.

For the TE 97‐ms PRESS, the 2.72‐ppm peak of cystathionine, which is attributed to two protons, is the highest among its signals at five locations and thus may largely be responsible for cystathionine estimation. The presence of the NAA and NAAG multiplets around 2.7 ppm may not cause considerable uncertainty in cystathionine estimation, since the strengths of the multiplets may be determined according to the strengths of their much larger singlets. The spectral overlap with the aspartate resonances could be concerning in cystathionine measurements since the major signals of the two metabolites overlap each other. The major signal polarities of the two metabolites are all positive, so their mutual effects on metabolite estimations occur in an inverse manner, in line with the negative correlation coefficients from LCModel. The degree of the interference of aspartate with cystathionine estimation would depend on the aspartate signal strengths. The concentration of aspartate, a major excitatory neurotransmitter [28], may reduce in brain tumors, similarly to the decreases in glutamate and NAA levels in brain tumors. When the aspartate level substantially decreases in tumors, as seen in Figure 7, the spectral overlap with aspartate may not cause considerable uncertainty in cystathionine measurements. An extensive spectral overlap is similarly present between 2HG and GABA in TE 97‐ms PRESS. Nonetheless, 2HG estimation by the PRESS method often provides an acceptable assessment of IDH status, as illustrated in this study (Figure 3E,F). This may be because GABA, a neurotransmitter, may decrease in brain tumors and thus have no substantial effect on 2HG measurements. It should be mentioned here that our estimates of aspartate and GABA were clearly lower than their normal‐brain concentrations and showed no considerable differences between the tumor groups divided by 1p/19q and IDH statuses. It follows that, despite their spectral overlaps with aspartate and GABA, altered levels of cystathionine and 2HG in brain tumors may be acceptably measurable by means of spectral fitting of TE 97‐ms PRESS spectra from patients.

A TE 113‐ms PRESS sequence is introduced in this paper as a tool for improving the signal specificity to cystathionine. Suppression of the aspartate signal between 2.62 and 2.82 ppm was achieved in favor of cystathionine detection by lengthening the PRESS TE_1_ and TE_2_ by 10 and 6 ms from the TE 97‐ms PRESS subecho times. The correlation coefficient between cystathionine and aspartate estimates in TE 113‐ms PRESS was much smaller compared with TE 97‐ms PRESS. The performance of the TE 113‐ms PRESS for cystathionine detection was further illustrated with 2D CSI of cystathionine in a glioma subject, which may be the first noninvasive imaging of cystathionine in a patient in vivo.

While the cystathionine signal difference between the TE 97‐ and 113‐ms PRESS schemes was minimal, the 16‐ms difference in PRESS TE gave rise to drastic alterations in the aspartate C3‐proton multiplet. This indicates that the density‐operator evolution during PRESS may be very different between the cystathionine and aspartate proton spins. For cystathionine, the ^2^CH, ^3^CH_2_, and ^4^CH_2_ proton spins may be modeled with an AMNPQ spin system. The C4‐proton spins P and Q, which are strongly coupled to the M and N spins and weakly coupled to the A spin, give rise to a cystathionine signal at 2.72 ppm. A PRESS simulation with the Philips slice‐selective 90° and 180° RF pulses of the current study indicated that, of the 60 single‐quantum coherences of P and Q spins, two inphase coherences (P_y_ and Q_y_) and six antiphase coherences (2P_x_M_z_, 2Q_x_M_z_, 2P_x_N_z_, 2Q_x_N_z_, 4P_y_M_z_N_z_, and 4Q_y_M_z_N_z_) may be largely responsible for the cystathionine 2.72‐ppm signal at TEs of 97 and 113 ms, as shown in Figure S4. When the T_2_ relaxation effect is ignored, the cystathionine peak is higher at TE = 113 ms than at TE = 97 ms, and the signal difference may be attributed to increases in P_y_ and Q_y_ coherences with the TE increase. In contrast, the TE 9‐ and 113‐ms PRESS multiplets of aspartate between 2.45 and 3.0 ppm may have noticeable signal contributions from all 16 single‐quantum coherences of the C3‐proton spins. When the aspartate ^3^CH_2_ and ^2^CH proton spins are modeled with an ABX spin system, the large positive and negative peaks at 2.74 and 2.66 ppm in TE 97‐ms PRESS may be largely attributed to four antiphase coherences (2A_y_X_z_, 2B_y_X_z_, 4A_y_B_z_X_z_, and 4B_y_A_z_X_z_). When the PRESS TE is changed to 113 ms, some coherences such as B_y_, B_x_, 2A_y_B_z_, and 4B_x_A_z_X_z_ get pronounced while the 2A_y_X_z_ and 2B_y_X_z_ coherences are decreased, resulting in marked reduction of the signal between 2.62 and 2.82 ppm while allowing for higher peaks at 2.58 and 2.89 ppm.

A recent study used a TE 45‐ms PRESS scheme for cystathionine detection [13], at which the aspartate C3‐proton multiplet was low. For our PRESS RF pulses, a TE 50‐ms PRESS option was also a candidate for cystathionine MRS, but we opted for the TE 113‐ms option for several reasons. First, the macromolecular signals are substantially attenuated during the 113‐ms TE, and consequently, a cystathionine signal can appear on a flat background. Second, while suppressing aspartate signal between 2.62 and 2.82 ppm, the TE 113‐ms PRESS gives rise to some sideband signals at 2.57 and 2.89 ppm, which can help to measure aspartate. Lastly, while the cystathionine C4‐proton multiplet at the TE of 113 ms exhibits a simple positive peak pattern, the cystathionine signal at TE of 50 ms consisted of positive and negative signals, whose signal amplitudes can be substantially reduced when B_0_ shimming is suboptimal.

Technical challenges associated with MRS measurements of 2HG and cystathionine were addressed as follows. First, although T2 signal loss is substantial in TE 97‐ and 113‐ms PRESS, these long‐TE MRS approaches may outperform short‐TE MRS because the metabolite signals are better resolved on top of a flat background in long‐TE spectra [16]. Second, the spectral overlap of the 2HG and lipid resonances at 2.25 ppm was resolved using a customized lipid basis set in spectral fitting, in which the lipid 2.25 ppm signal was predetermined with reference to the strong 0.9‐ppm lipid resonance [20]. Third, since all metabolite levels are altered in brain tumors, metabolites were quantified with reference to water. The water signal was recorded with long TR and short TE to minimize the effects of water T1 and T2 relaxations, which are very different between brain tumors and normal brain. Lastly, every effort was made to position a voxel completely within the lesion (e.g., T2‐FLAIR hyperintense volume), which is important for minimizing signals from nontumoral volumes.

Several limitations are present in the current study. First, the small sample size is a pitfall. Our findings may require validation in a large number of cohort subjects to have sufficient statistical power. In particular, the TE 113‐ms PRESS scheme needs to be tested in additional patients. Second, since the T1 and T2 of large molecules such as glutathione [29, 30] as well as macromolecules [31] are relatively short, the relaxation times of cystathionine could also be relatively short. Accurate measurement of cystathionine requires correction for T1 and T2 relaxation effects on the cystathionine signals, which are beyond the scope of the present study. Since the metabolite relaxation times and their ratios could be distinctly different between brain and aqueous solutions [24, 32], correcting for in vivo relaxation effects using phantom relaxation times was not pursued in the present study. Third, while the water content difference between tumor and healthy brain was ignored in our metabolite quantification, measurement of the water content using proton‐density imaging would confer an accurate assessment of tumor metabolite levels since the water content in brain tumor appears to be higher by approximately 20% compared with normal white‐matter brain [20, 33]. Fourth, although the tumor metabolism associated with cystathionine elevation in 1p/19q codeleted gliomas was extensively investigated in a prior study [25], further research on cystathionine biology would be additive and valuable. A tumor tissue study on cystathionine metabolism was beyond the scope of this technical MRS study. Lastly, since the signal pattern and strength of J‐coupled spin metabolites depend on the slice‐selective refocusing RF pulses, the optimized subecho times of the TE 97‐ and 113‐ms PRESS schemes may be fully applicable to the 180° RF pulses used in the present study, needing some adjustments in inter‐RF pulse timings for the PRESS sequences having other types of refocusing pulses.

## 5 Conclusion

Our TE 97‐ms PRESS data confirmed that cystathionine is elevated in patients with 1p/19q codeleted gliomas, as reported in prior studies [7, 8]. While 2HG estimation by the same MRS method resulted in complete distinction between IDH‐mutant and IDH‐wildtype tumors, the sensitivity and specificity of the cystathionine estimates to 1p/19q status were much lower than 100%. This may be largely because the biological specificity of cystathionine elevation to 1p/19q codeletion is lower compared to that of 2HG elevation to IDH mutation. It follows that only high estimates of cystathionine (> 1.3 mM) provide a biomarker of 1p/19q codeleted gliomas. TE 113‐ms PRESS provided suppression of the aspartate multiplet between 2.62 and 2.82 ppm while maintaining the cystathionine peak at 2.72 ppm, thereby improving the signal specificity of cystathionine in brain MRS.

## Author Contributions

**Changho Choi:** writing – original draft, conceptualization, methodology, software, formal analysis, visualization, supervision, funding acquisition. **Mai Huynh:** writing – review and editing, investigation, resources. **Zoltan Kovacs:** writing – review and editing, investigation, resources, supervision. **William J. Behof:** writing – review and editing, investigation, resources. **Wellington Pham:** writing – review and editing, investigation, resources, supervision. **Sandeep K. Ganji:** writing – review and editing, methodology, software, data curation, visualization. **Zhongxu An:** writing – review and editing, methodology, software, data curation, visualization. **Toral R. Patel:** writing – review and editing, resources, project administration. **Elizabeth A. Maher:** writing – review and editing, resources, project administration. **Julia D. Berry:** writing – review and editing, validation, project administration. **Bret C. Mobley:** writing – review and editing, validation, project administration. **Larry T. Davis:** writing – review and editing, validation, project administration. **Colin D. McKnight:** writing – review and editing, validation, project administration. **Sumit Pruthi:** writing – review and editing, validation, project administration. **Ryan T. Merrell:** writing – review and editing, validation, project administration. **Alexander C. Mohler:** writing – review and editing, validation, project administration. **Patrick D. Kelly:** writing – review and editing, validation, project administration. **Reid C. Thompson:** writing – review and editing, validation, project administration, funding acquisition.

## Funding

This work was supported by the National Institutes of Health (grant numbers R01CA184584, S10OD021771, and UL1TR002243) and by the Cancer Prevention and Research Institute of Texas (grant numbers RP130427 and RP200456). We acknowledge support from VICC, VUIIS, VICTR, and the Departments of Radiology and Radiological Sciences and Neurological Surgery of Vanderbilt University Medical Center.

## Conflicts of Interest

The authors declare no conflicts of interest.

## Supporting information


**Figure S1:** (A) The number of PRESS signal averages is plotted vs. voxel size for the 38 patients of the present study. (B) The tCho (total choline) SNR (singlet height to noise ratio) is plotted vs. voxel size for the 38 patients of the present study. The noise level was calculated as the standard deviation of the LCModel‐returned residuals between 0.2 and 4.0 ppm.
**Figure S2:** In vivo TE 97‐ms PRESS spectra, acquired from 20 × 20 × 20‐mm^3^ voxels positioned in the white‐matter regions of four healthy subjects (128 averages, TR 2 s), are shown together with LCModel‐returned fit, residuals, and metabolite signals and concentration estimates. The LCModel estimates of the metabolite signals were normalized to TR 20‐s and TE 14‐ms water signal and subsequently the metabolite‐to‐water signal ratios were scaled to mM values with reference to tCr at 6 mM. As a result, the mean tCr concentration estimate was 6 mM (6.0 ± 0.4 mM). A proportional coefficient was obtained from this scaling process and used for calculating the mM concentrations of metabolites in brain tumors. CST, cystathionine.
**Figure S3:** In vivo TE 97‐ms PRESS spectra from 38 glioma patients (P1–P38) are presented in the order of the patients in Figure 3C. Each subfigure is titled the patient number and the IDH and 1p/19q status. Within each subfigure, top to bottom, the tCho FWHM, voxel size, number of signal averages, and tCho SNR. TR was 2 s in all scans. Here, the tCho FWHM was measured from the sum of the LCModel‐returned tCho signals (GPC and PCh). The tCho SNR was calculated as a ratio of the tCho peak amplitude with respect to the standard deviation of the LCModel‐returned residuals between 0.2 and 4.0 ppm. For the 38 spectra, the tCho FWHM, voxel size, signal averaging, and tCho SNR were 6.2 ± 1.2 Hz (range 4.6–9.4 Hz), 6.8 ± 2.0 mL (range 2.1–11.4 mL), 210 ± 135 (range 128–768), and 110 ± 42 (range 33–204), respectively. Abbreviations: IDHm = IDH mutated; IDHw = IDH wildtype; Codel = 1p/19q codeleted; Noncodel = 1p/19q noncodeleted.
**Figure S4:** (A) Calculated TE 97‐ and 113‐ms PRESS signals of the cystathionine C4‐proton spins are shown together with the individual signals of 15 coherence terms. The TE 97‐ and 113‐ms PRESS signals between 2.42 and 3.02 ppm are displayed on top of each other, ignoring T2 relaxation effects. Here, the cystathionine's ^2^CH, ^3^CH_2_, and ^4^CH_2_ proton spins are modeled with an AMNPQ spin system. Of the entire 60 inphase and antiphase terms of the P and Q spins (i.e., ^4^CH_2_ proton spins), 15 coherence terms were selected, whose signal amplitudes were higher than 2% of the ^4^CH_2_ proton peak at TE = 97 ms. (B) Calculated TE 97‐ and 113‐ms PRESS signals of the aspartate C3‐proton spins are shown together with the individual signals of 16 coherence terms. The TE 97‐ and 113‐ms PRESS signals between 2.42 and 3.02 ppm are displayed on top of each other, ignoring T2 relaxation effects. Here, the aspartate's ^3^CH_2_ and ^2^CH proton spins are modeled with an ABX spin system. Individual signals of the entire 16 inphase and antiphase coherences of the A and B spins (i.e., ^3^CH_2_ proton spins) are displayed, each of which was higher than 2% of the ^3^CH_2_ proton peak at TE = 97 ms. For both cystathionine and aspartate, the spectra were broadened to singlet FWHM of 5 Hz.

## Data Availability

The data that support the findings of this study are available on request from the corresponding author. The data are not publicly available due to privacy or ethical restrictions.

## References

[1] D. N. Louis , A. Perry , P. Wesseling , et al., “The 2021 WHO Classification of Tumors of the Central Nervous System: A Summary,” Neuro‐Oncology 23 (2021): 1231–1251. 34185076 10.1093/neuonc/noab106 PMC8328013

[2] D. J. Brat , R. G. Verhaak , K. D. Aldape , et al., “Comprehensive, Integrative Genomic Analysis of Diffuse Lower‐Grade Gliomas,” New England Journal of Medicine 372 (2015): 2481–2498. 26061751 10.1056/NEJMoa1402121 PMC4530011

[3] L. Dang , D. W. White , S. Gross , et al., “Cancer‐Associated IDH1 Mutations Produce 2‐Hydroxyglutarate,” Nature 462 (2009): 739–744. 19935646 10.1038/nature08617 PMC2818760

[4] C. Choi , S. K. Ganji , R. J. Deberardinis , et al., “2‐Hydroxyglutarate Detection by Magnetic Resonance Spectroscopy in IDH‐Mutated Patients With Gliomas,” Nature Medicine 18 (2012): 624–629. 10.1038/nm.2682 PMC3615719 22281806

[5] W. B. Pope , R. M. Prins , M. Albert Thomas , et al., “Non‐Invasive Detection of 2‐Hydroxyglutarate and Other Metabolites in IDH1 Mutant Glioma Patients Using Magnetic Resonance Spectroscopy,” Journal of Neuro‐Oncology 107 (2012): 197–205. 22015945 10.1007/s11060-011-0737-8 PMC3650613

[6] O. C. Andronesi , G. S. Kim , E. Gerstner , et al., “Detection of 2‐Hydroxyglutarate in IDH‐Mutated Glioma Patients by In Vivo Spectral‐Editing and 2D Correlation Magnetic Resonance Spectroscopy,” Science Translational Medicine 4 (2012): 116ra114. 10.1126/scitranslmed.3002693 PMC3720836 22238332

[7] F. Branzoli , A. L. Di Stefano , L. Capelle , et al., “Highly Specific Determination of IDH Status Using Edited In Vivo Magnetic Resonance Spectroscopy,” Neuro‐Oncology 20 (2018): 907–916. 29126125 10.1093/neuonc/nox214 PMC6007442

[8] F. Branzoli , R. Liserre , D. K. Deelchand , et al., “Neurochemical Differences Between 1p/19q Codeleted and Noncodeleted IDH‐Mutant Gliomas by In Vivo MR Spectroscopy,” Radiology 308 (2023): e223255. 37668523 10.1148/radiol.223255 PMC10546286

[9] F. Branzoli , D. K. Deelchand , M. Sanson , S. Lehericy , and M. Marjanska , “In Vivo (1) H MRS Detection of Cystathionine in Human Brain Tumors,” Magnetic Resonance in Medicine 82 (2019): 1259–1265. 31131476 10.1002/mrm.27810 PMC6626581

[10] V. Govind , “1H‐NMR Chemical Shifts and Coupling Constants for Brain Metabolites,” eMagRes 5 (2016): 1347–1362

[11] C. H. Suh , H. S. Kim , S. C. Jung , C. G. Choi , and S. J. Kim , “2‐Hydroxyglutarate MR Spectroscopy for Prediction of Isocitrate Dehydrogenase Mutant Glioma: A Systemic Review and Meta‐Analysis Using Individual Patient Data,” Neuro‐Oncology 20 (2018): 1573–1583. 30020513 10.1093/neuonc/noy113 PMC6231199

[12] A. Bhandari , C. Sharma , M. Ibrahim , M. Riggs , R. Jones , and A. Lasocki , “The Role of 2‐Hydroxyglutarate Magnetic Resonance Spectroscopy for the Determination of Isocitrate Dehydrogenase Status in Lower Grade Gliomas Versus Glioblastoma: A Systematic Review and Meta‐Analysis of Diagnostic Test Accuracy,” Neuroradiology 63 (2021): 1823–1830. 33811494 10.1007/s00234-021-02702-1

[13] M. Zhou , Z. Nie , J. Zhao , et al., “Optimization and Validation of Echo Times of Point‐Resolved Spectroscopy for Cystathionine Detection in Gliomas,” Cancer Imaging 24 (2024): 118. 39223589 10.1186/s40644-024-00764-x PMC11367870

[14] J. E. Eckel‐Passow , D. H. Lachance , A. M. Molinaro , et al., “Glioma Groups Based on 1p/19q, IDH, and TERT Promoter Mutations in Tumors,” New England Journal of Medicine 372 (2015): 2499–2508. 26061753 10.1056/NEJMoa1407279 PMC4489704

[15] R. B. Thompson and P. S. Allen , “Sources of Variability in the Response of Coupled Spins to the PRESS Sequence and Their Potential Impact on Metabolite Quantification,” Magnetic Resonance in Medicine 41 (1999): 1162–1169. 10371448 10.1002/(sici)1522-2594(199906)41:6<1162::aid-mrm12>3.0.co;2-n

[16] C. Choi , S. Ganji , K. Hulsey , et al., “A Comparative Study of Short‐ and Long‐TE (1)H MRS at 3 T for In Vivo Detection of 2‐Hydroxyglutarate in Brain Tumors,” NMR in Biomedicine 26 (2013): 1242–1250. 23592268 10.1002/nbm.2943 PMC3733061

[17] T. Shiraiwa , K. Nakagawa , N. Kanemoto , T. Kinda , and H. Yamamoto , “Synthesis of Optically Active Homocysteine From Methionine and Its Use in Preparing Four Stereoisomers of Cystathionine,” Chemical & Pharmaceutical Bulletin (Tokyo) 50 (2002): 1081–1085. 10.1248/cpb.50.1081 12192140

[18] R. J. Ogg , P. B. Kingsley , and J. S. Taylor , “WET, a T1‐ and B1‐Insensitive Water‐Suppression Method for In Vivo Localized 1H NMR Spectroscopy,” Journal of Magnetic Resonance. Series B 104 (1994): 1–10. 8025810 10.1006/jmrb.1994.1048

[19] S. W. Provencher , “Estimation of Metabolite Concentrations From Localized In Vivo Proton NMR Spectra,” Magnetic Resonance in Medicine 30 (1993): 672–679. 8139448 10.1002/mrm.1910300604

[20] P. Askari , I. E. Dimitrov , S. K. Ganji , et al., “Spectral Fitting Strategy to Overcome the Overlap Between 2‐Hydroxyglutarate and Lipid Resonances at 2.25 ppm,” Magnetic Resonance in Medicine 86 (2021): 1818–1828. 33977579 10.1002/mrm.28829 PMC8295210

[21] V. Tiwari , Z. An , Y. Wang , and C. Choi , “Distinction of the GABA 2.29 ppm Resonance Using Triple Refocusing at 3 T In Vivo,” Magnetic Resonance in Medicine 80 (2018): 1307–1319. 29446149 10.1002/mrm.27142 PMC6092256

[22] E. Hattingen , P. Raab , K. Franz , et al., “Prognostic Value of Choline and Creatine in WHO Grade II Gliomas,” Neuroradiology 50 (2008): 759–767. 18523762 10.1007/s00234-008-0409-3

[23] S. K. Ganji , A. Banerjee , A. M. Patel , et al., “T2 Measurement of J‐Coupled Metabolites in the Human Brain at 3T,” NMR in Biomedicine 25 (2012): 523–529. 21845738 10.1002/nbm.1767 PMC3852663

[24] A. Madan , S. K. Ganji , Z. An , et al., “Proton T2 Measurement and Quantification of Lactate in Brain Tumors by MRS at 3 Tesla In Vivo,” Magnetic Resonance in Medicine 73 (2015): 2094–2099. 25046359 10.1002/mrm.25352 PMC4286522

[25] F. Branzoli , C. Pontoizeau , L. Tchara , et al., “Cystathionine as a Marker for 1p/19q Codeleted Gliomas by In Vivo Magnetic Resonance Spectroscopy,” Neuro‐Oncology 21 (2019): 765–774. 30726924 10.1093/neuonc/noz031 PMC6556848

[26] A. K. Akobeng , “Understanding Diagnostic Tests 3: Receiver Operating Characteristic Curves,” Acta Paediatrica 96 (2007): 644–647. 17376185 10.1111/j.1651-2227.2006.00178.x

[27] M. Mizoguchi , K. Yoshimoto , X. Ma , et al., “Molecular Characteristics of Glioblastoma With 1p/19q Co‐Deletion,” Brain Tumor Pathology 29 (2012): 148–153. 22736234 10.1007/s10014-012-0107-z

[28] G. Siegel , B. Agranoff , R. Albers , S. Fisher , and M. Uhler , Basic Neurochemistry: Molecular, Cellular and Medical Aspects (Lippincott‐Raven, 1999).

[29] I. Y. Choi and P. Lee , “Doubly Selective Multiple Quantum Chemical Shift Imaging and T(1) Relaxation Time Measurement of Glutathione (GSH) in the Human Brain In Vivo,” NMR in Biomedicine 26 (2013): 28–34. 22730142 10.1002/nbm.2815 PMC3465620

[30] G. Choi , S. Kim , R. Noeske , et al., “Evaluation of Glutathione T(2) in the Human Brain Using J‐Difference MRS at 3 T: Multicenter Multivendor Study,” NMR in Biomedicine 38 (2025): e5313. 39776150 10.1002/nbm.5313 PMC11707642

[31] K. L. Behar , D. L. Rothman , D. D. Spencer , and O. A. Petroff , “Analysis of Macromolecule Resonances in 1H NMR Spectra of Human Brain,” Magnetic Resonance in Medicine 32 (1994): 294–302. 7984061 10.1002/mrm.1910320304

[32] C. Choi , N. J. Coupland , P. P. Bhardwaj , et al., “T2 Measurement and Quantification of Glutamate in Human Brain In Vivo,” Magnetic Resonance in Medicine 56 (2006): 971–977. 17029225 10.1002/mrm.21055

[33] A. M. Oros‐Peusquens , R. Loucao , M. Zimmermann , K. J. Langen , and N. J. Shah , “Methods for Molecular Imaging of Brain Tumours in a Hybrid MR‐PET Context: Water Content, T_2_*, Diffusion Indices and FET‐PET,” Methods 130 (2017): 135–151. 28774682 10.1016/j.ymeth.2017.07.025

