# Supplementary material for: Magnetic Resonance Spectroscopy of Cystathionine and 2‐Hydroxyglutarate in Brain Tumors
Source: NMR Biomed. 2025 Nov 25;39(1):e70181. doi: 10.1002/nbm.70181 (PMC12646717; doi:10.1002/nbm.70181)

SUPPORTING INFORMATION

Figure S1. (**A**) The number of PRESS signal averages is plotted vs voxel size for the 38 patients of the present study. (**B**) The tCho (total choline) SNR (singlet height to noise ratio) is plotted vs voxel size for the 38 patients of the present study. The noise level was calculated as the standard deviation of the LCModel-returned residuals between 0.2 - 4.0 ppm.

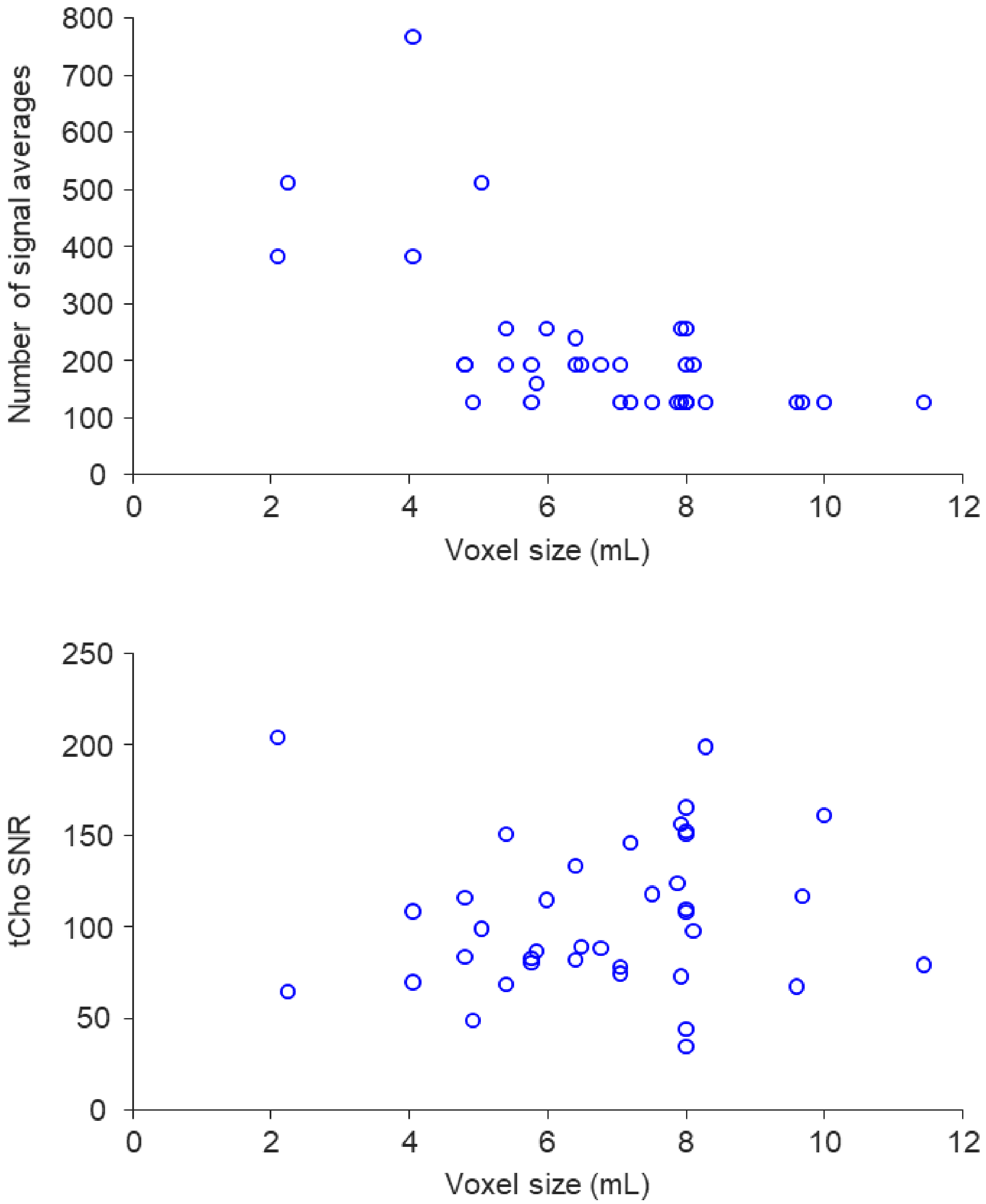

Figure S2. In vivo TE 97 ms PRESS spectra, acquired from 20x20x20 mm^3 voxels positioned in the white matter regions of four healthy subjects (128 averages, TR 2 s), are shown together with LCModel-returned fit, residuals, and metabolite signals and concentration estimates. The LCModel estimates of the metabolite signals were normalized to TR 20 s and TE 14 ms water signal and subsequently the metabolite-to-water signal ratios were scaled to mM values with reference to tCr at 6 mM. As a result, the mean tCr concentration estimate was 6 mM (6.0±0.4 mM). A proportional coefficient was obtained from this scaling process and used for calculating the mM concentrations of metabolites in brain tumors. CST, cystathionine.

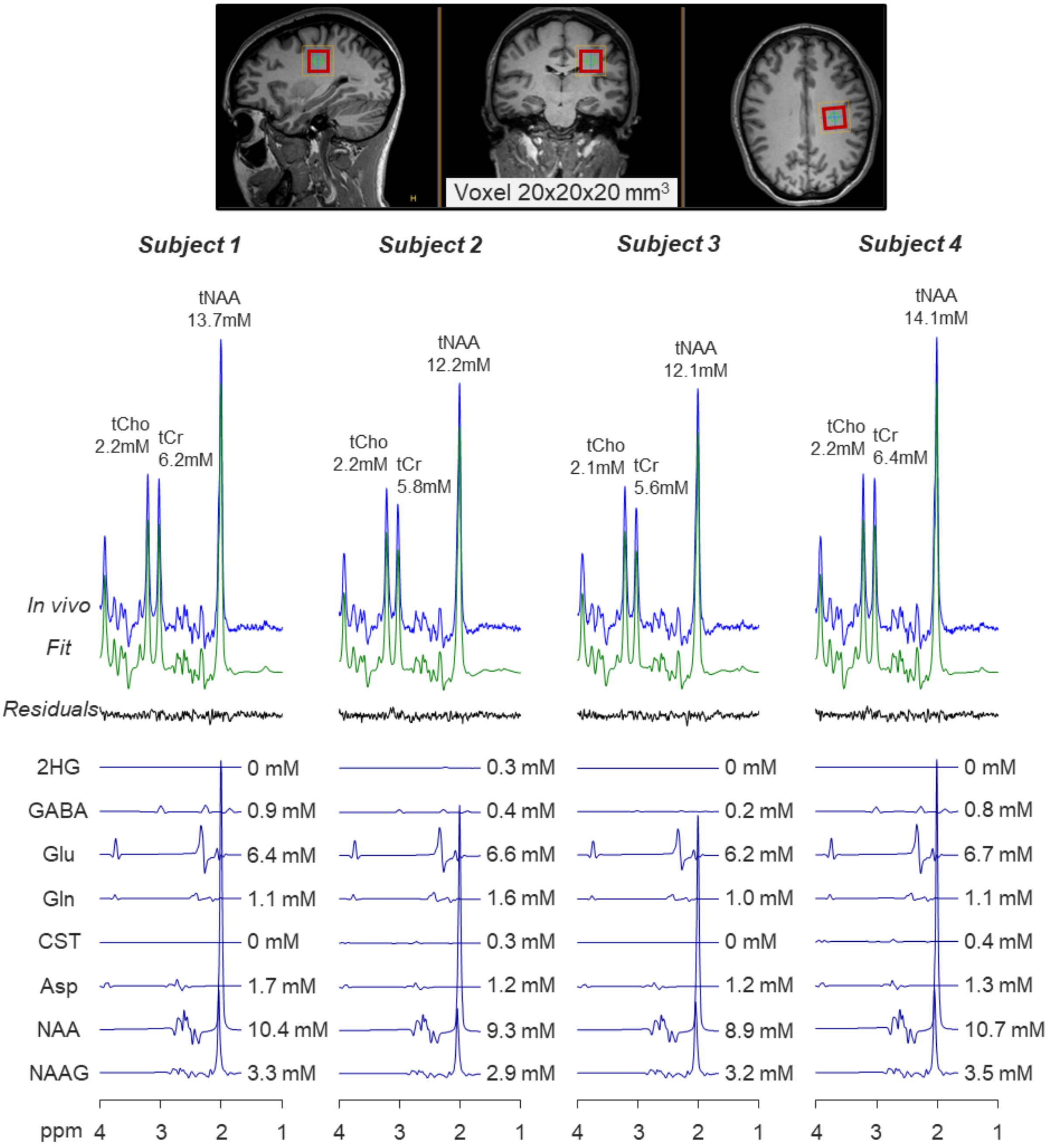

Figure S3. In vivo TE 97 ms PRESS spectra from 38 glioma patients (P1 - P38) are presented in the order of the patients in Figure 3C. Each subfigure is titled the patient number and the IDH and 1p/19q status. Within each subfigure, top to bottom, the tCho FWHM, voxel size, number of signal averages, and tCho SNR. TR was 2 s in all scans. Here the tCho FWHM was measured from the sum of the LCModel-returned tCho signals (GPC and PCh). The tCho SNR was calculated as a ratio of the tCho peak amplitude with respect to the standard deviation of the LCModel-returned residuals between 0.2 - 4.0 ppm. For the 38 spectra, the tCho FWHM, voxel size, signal averaging, and tCho SNR were 6.2±1.2 Hz (range 4.6 - 9.4 Hz), 6.8±2.0 mL (range 2.1 - 11.4 mL), 210±135 (range 128 - 768), and 110±42 (range 33 - 204), respectively. Abbreviations: IDHm = IDH mutated; IDHw = IDH wildtype; Codel = 1p/19q codeleted; Noncodel = 1p/19q noncodeleted.

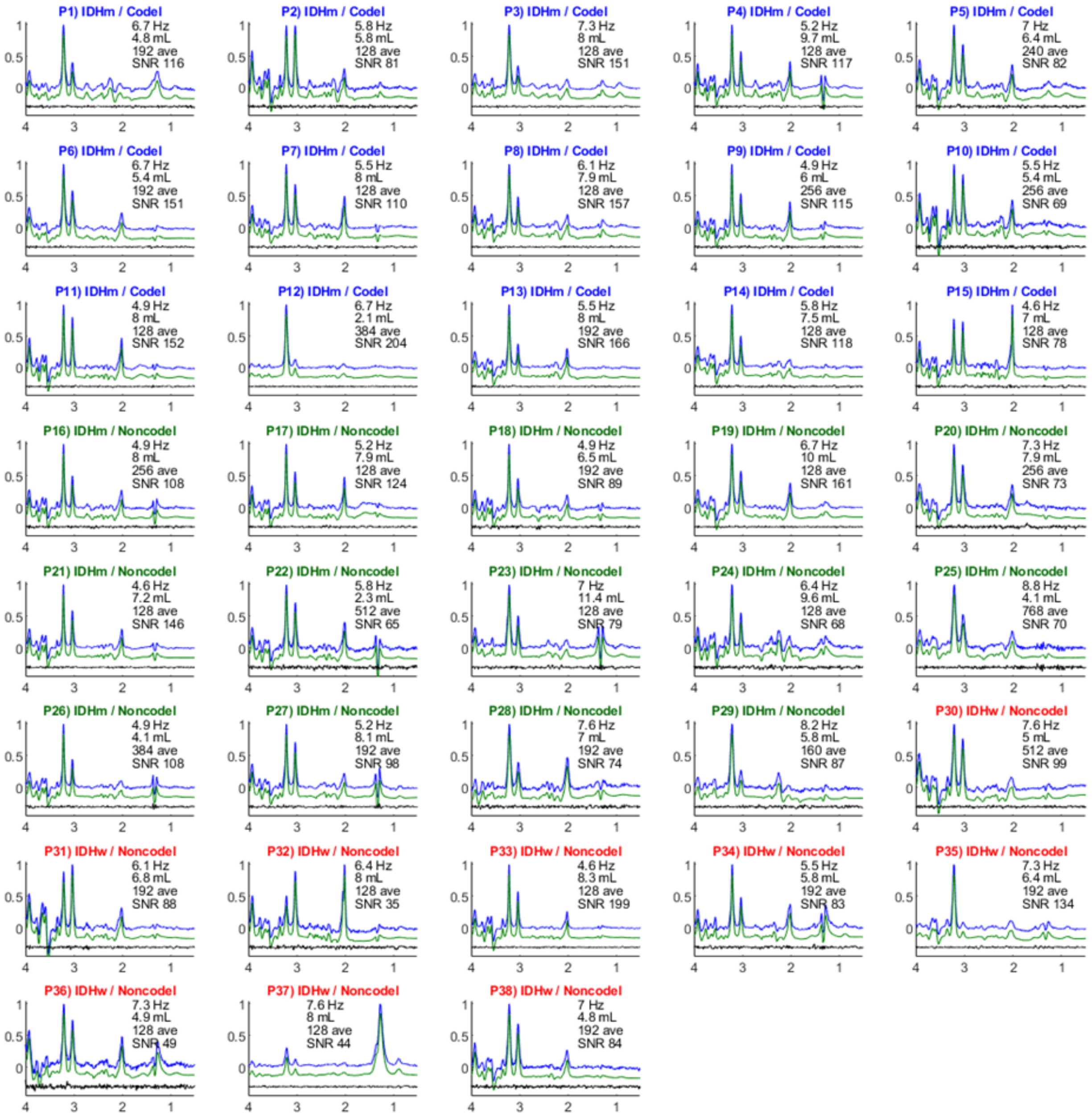

Figure S4. (**A**) Calculated TE 97 and 113 ms PRESS signals of the cystathionine C4-proton spins are shown together with the individual signals of 15 coherence terms. The TE 97 and 113 ms PRESS signals between 2.42 - 3.02 ppm are displayed on top of each other, ignoring T2 relaxation effects. Here, the cystathionine's ^{2}CH, ^{3}CH$_2$, and ^{4}CH$_2$ proton spins are modeled with an AMNPQ spin system. Of the entire 60 inphase and antiphase terms of the P and Q spins (i.e., ^{4}CH$_2$ proton spins), 15 coherence terms were selected, whose signal amplitudes were higher than 2% of the ^{4}CH$_2$ proton peak at TE = 97 ms. (**B**) Calculated TE 97 and 113 ms PRESS signals of the aspartate C3-proton spins are shown together with the individual signals of 16 coherence terms. The TE 97 and 113 ms PRESS signals between 2.42 - 3.02 ppm are displayed on top of each other, ignoring T2 relaxation effects. Here, the aspartate's ^{3}CH$_2$ and ^{2}CH proton spins are modeled with an ABX spin system. Individual signals of the entire 16 inphase and antiphase coherences of the A and B spins (i.e., ^{3}CH$_2$ proton spins) are displayed, each of which was higher than 2% of the ^{3}CH$_2$ proton peak at TE = 97 ms. For both cystathionine and aspartate, the spectra were broadened to singlet FWHM of 5 Hz.

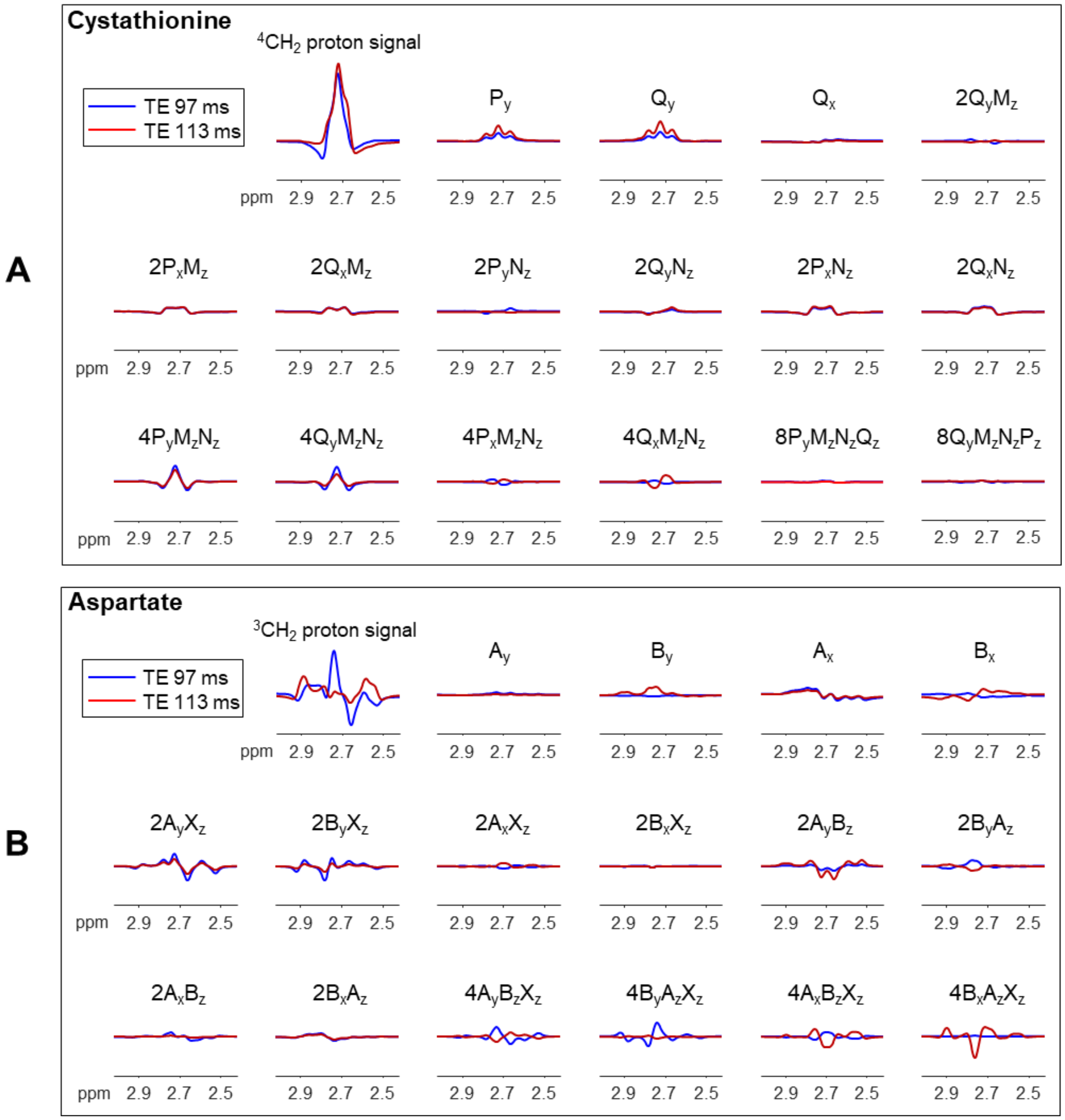

Supplement: Supplementary file 1 — Figure S1: (A) The number of PRESS signal averages is plotted vs. voxel size for the 38 patients of the present study. (B) The tCho (total choline) SNR (singlet height to noise ratio) is plotted vs. voxel size for the 38 patients of the present study. The noise level was calculated as the standard deviation of the LCModel‐returned residuals between 0.2 and 4.0 ppm. Figure S2: In vivo TE 97‐ms PRESS spectra, acquired from 20 × 20 × 20‐mm3 voxels positioned in the white‐matter regions of four healthy subjects (128 averages, TR 2 s), are shown together with LCModel‐returned fit, residuals, and metabolite signals and concentration estimates. The LCModel estimates of the metabolite signals were normalized to TR 20‐s and TE 14‐ms water signal and subsequently the metabolite‐to‐water signal ratios were scaled to mM values with reference to tCr at 6 mM. As a result, the mean tCr concentration estimate was 6 mM (6.0 ± 0.4 mM). A proportional coefficient was obtained from this scaling process and used for calculating the mM concentrations of metabolites in brain tumors. CST, cystathionine. Figure S3: In vivo TE 97‐ms PRESS spectra from 38 glioma patients (P1–P38) are presented in the order of the patients in Figure 3C. Each subfigure is titled the patient number and the IDH and 1p/19q status. Within each subfigure, top to bottom, the tCho FWHM, voxel size, number of signal averages, and tCho SNR. TR was 2 s in all scans. Here, the tCho FWHM was measured from the sum of the LCModel‐returned tCho signals (GPC and PCh). The tCho SNR was calculated as a ratio of the tCho peak amplitude with respect to the standard deviation of the LCModel‐returned residuals between 0.2 and 4.0 ppm. For the 38 spectra, the tCho FWHM, voxel size, signal averaging, and tCho SNR were 6.2 ± 1.2 Hz (range 4.6–9.4 Hz), 6.8 ± 2.0 mL (range 2.1–11.4 mL), 210 ± 135 (range 128–768), and 110 ± 42 (range 33–204), respectively. Abbreviations: IDHm = IDH mutated; IDHw = IDH wildtype; Codel = 1p/19q codeleted; N [file NBM-39-e70181-s001.pdf]
